# Lose the label: Messaging effects on Democrats’ perceptions of individuals with criminal records and representation

**DOI:** 10.1371/journal.pone.0355933

**Published:** 2026-08-27

**Authors:** Jessie Harney

**Affiliations:** Department of Political Science, Colorado State University, Fort Collins, Colorado, United States of America; Yeditepe University, TÜRKIYE

## Abstract

During the 2024 U.S. presidential election, Democratic campaign advertisements frequently labeled Trump as a “convicted criminal,” invoking stigmatizing language with empirically known, negative consequences for public perceptions of individuals with criminal records. Leaning upon social categorization theory, propagating such labels on Trump may thereby spillover, impacting constituents’ perspectives of individuals with records more broadly. To test for the possibility of stigmatizing language spillover, this preliminary study employs a survey experiment testing exposure to the Party’s advertisement, labeling Trump as a “convicted criminal” (*n* = 814) relative to no advertisement (*n* = 813), on Democrats’ perceptions of individuals with records and several dimensions of perceived representation. Results suggest that exposure to the Trump advertisement with the “convicted criminal” label had significant, negative impacts on Democrats’ perceptions of individuals with records, but improved perceived feelings of representation. Importantly, the effect of a counter-stereotype’s ability to mitigate these effects was also tested, randomly assigning some participants to receive the perspective of a formerly system-involved state representative before the advertisement (*n* = 812). Findings suggest that the counter-stereotype vastly ameliorated the negative effects of the advertisement on Democrats’ perceptions of individuals with criminal records. While well-powered and aptly-timed field experiments are needed to confirm and extend these findings, utilizing political messaging that elevates dedicated, justice-involved voices within the party rather than rely on stigmatizing attacks should be strongly considered and tested.

## Introduction

On May 30, 2024, Donald J. Trump was convicted of 34 felony crimes related to his attempts to illegally influence his 2016 presidential election [1]. In the days, weeks, and months that followed, labels of Trump being a ‘convicted criminal,’ ‘felon’, or other terms used to schematize Trump as belonging to a particular group – individuals with a criminal record, or more specifically a felony record – proliferated across social media, television news, and other media platforms. In fact, according to ProQuest, there were 297 unique articles published on that day or the following resulting from a “Trump felony” search, 23 of which directly referenced him as a ‘felon’ or ‘convicted felon’ in the *title of the article alone* [2]. Subsequently, during the 2024 presidential election, the label of ‘convicted criminal’, ‘felon’, and other stigmatizing terms were applied to Trump, amplified through political advertisements run by the Democratic Party [3].

Though acknowledgement of Trump’s convictions by the Democratic Party was likely unavoidable and in fact, necessary, the use of stigmatizing terms was not. The more negative angle for campaign advertisements and rhetoric is important to note: prior evidence on the effects of negative campaign advertisements suggests that they tend to be utilized less by those leading in the polls [4,5] and that Democrats tend to respond more to positive ads, compared to Republicans [6]. Additionally, meta-analytic evidence does not empirically support the use of negative campaign advertisements to aid election success [7]. Extant literature highlights the causal impact of propagation of labels, such as ‘criminal’, to significantly worsen perceptions of individuals with records, relative to more humanizing, person-first language, such as ‘a person with a criminal record’ [8–10]. Additionally, the extent to which individuals feel angry towards those with criminal records has been found to be associated with greater support for *more punitive* justice system policy [11]. Thus, even if the intention of the advertisements depicting Trump as a “convict,” “criminal,” or “felon” were intended for purpose of impacting *solely* the likelihood of his ability to win the presidential election (e.g., encouraging action and anger towards Trump), social categorization theory [12–15] would suggest that the effects of utilizing this label likely spilled over.

Specifically, social categorization theory articulates the implications and *process* of how individuals come to assign and distinguish individuals into groups, with much of the theory and evidence pointing to the importance of category salience and the role of group exemplars and prototypes [16]. Greater salience of a category – in psychological literature, often perceived sex and racial/ethnic identity – tend to be operationalized and activated more easily, given the category’s saliency [17–19]. Particularly when an individual’s level of exposure to a group or category is more limited, they may be more likely to lean upon perceptions of group exemplars (i.e., real individuals who are members of this group) to fill in the gaps *for other members of that group*, with saliency of a group member increasing the propensity for that member to be an exemplar [20,21]. Thus, in this context, once the label of ‘felon’ and ‘convicted criminal’ was applied to Donald J. Trump – and done so quite routinely and saliently – theory and evidence would predict greater homogenization and spillover perceptions from Trump, such that perceptions of individuals with records would be *worsened* amongst Democrats after exposure to the advertisement, including its affixed, “convicted criminal” label.

Even if Democrats, on average, may be less amenable to punitive justice system policy and less likely to perceive that crime is on the rise when it may not be [22], policy support is distinct from *perceptions of individuals* who would be directly impacted by these policies. Perhaps the clearest example of this possibility in literature on the dynamics between policy support and perceptions of its beneficiaries is that of the social safety net. Public support for the social safety net is quite strong – estimates suggest that a majority of U.S. residents support universal health insurance, extension of unemployment benefits, and other safety-net related policies [23] – yet, this does not necessarily coincide with positive perceptions of those who utilize these programs. In fact, highly racist and sexist tropes have proliferated and been utilized as tools to influence public opinion – e.g., propagating = terms such as ‘welfare queens’ and stereotypes related to work ethic [24] – which stigmatizes these programs [25]. Psychologically, individuals may be engaging in motivated reasoning, as rhetoric aligning with prior beliefs about the beneficiaries (i.e., they must ‘deserve’ or ‘work for’ their benefits), is unchallenged and thus, why *elevating* administrative burden may *strengthen* perceived deservingness of the social safety net, including for those who are system-impacted [25,26].

Additionally, in a not-so-distant past, Democratic presidents championed punitive policies, such as the 1994 crime bill, amplifying mass incarceration’s impact through expansion of prison building and implementation of mandatory minimums [27], during a time in which 42% of the U.S. reported crime as the *most* important problem facing the U.S., compared to just about 3% in recent years [28]. Media portrayals of category members – when they are made to be more salient – are, more often than not, quite negatively depicted (e.g., mugshots and more reporting on the exceptionally horrific or violent crimes). In combination with the increased salience and potentially greater reliance on salient members as exemplars, such as Donald J. Trump, there could still be disconnect between policy support and public perceptions of those who are most immediately impacted by these policies.

There are likely several reasons – either in isolation or in combination – for which this approach was employed by the Democratic Party: a) to encourage those who were marginal on *who* they would vote for to vote for Vice President Harris; and/or b) to encourage those who were marginal on *if* they would vote to do so (presumably voting for Vice President Harris); and/or c) to encourage involvement in the campaign amongst the Democratic party base, particularly for strong Democrats [29]. A considerable body of evidence suggests that invoking an emotional response may encourage political support, which may downplay focusing on representation regarding policy positions, leaning instead into heightening in-group ties and fostering affective polarization [30–32]. For example, one study found that anger-inducing political advertisements bolstered support and encouraged action specifically amongst members of the party producing the advertisement [33].

However, this is not necessarily concrete, in that alignment of policy standpoints has been shown to be crucial to feelings of representation [34–36]. In fact, experimental evidence suggests that policy positions and elected officials taking action tend to have a stronger causal effect on feelings of being represented, rather than affect [37]. Additionally, empirical political messaging and advertising tend to suggest that they produce little to no demonstrable effects, with few exceptions [38], and recent robust experimental work suggests that affective polarization may not have meaningful impact on political outcomes, though may have some implications for interpersonal dynamics, particularly on dimensions of connection with members of party out-groups [39].

While support for criminal justice system reform has become considerably bipartisan in the recent past [40,41], Democrats’ policy preferences tend to be much less supportive of punitive justice system policy than Republicans [42]. Thus, stigmatizing those with records through political advertisements has the propensity to discourage Democrats from feeling connected with or represented by their Party, potentially instigating a backfire effect, though this is also not guaranteed. Alternatively, one presumably unexplored messaging intervention in the 2024 election – though heavily supported by political science and psychological literature – is perspective-getting [42–44]. Perspective-getting, or being provided with the perspective of another, has been shown to significantly increase feelings of empathy for members of an out-group [45,46], including incarcerated individuals [47]. Thus, sharing political messaging highlighting dedicated, system-impacted representatives that may provide both their perspective (i.e., encouraging empathy, positive emotions, and understanding) as well as one that signals their policy alignment (i.e., dedication to justice system reform), would be well-suited to addressing both the affective and policy representation avenues for bolstering support.

Increasingly, formerly system-involved folks dedicated to structural and systemic reform have broken down barriers to representation, entering various levels of government and political offices [48]. Take for example, Jovan Jackson, an assemblymember (D), and likely, the first formerly incarcerated person to serve as a state legislator in Nevada. Jackson’s advocacy in and outside of political office, has been consistent since his release in 2018, such as his efforts in organizing to help pass AB431 in Nevada, ensuring that formerly incarcerated people have their ability to vote automatically restored after release [49]. Assemblymember Jackson is one of a growing number of formerly system-involved representatives motivated to usher forward system change, such as Tarra Simmons, the first formerly incarcerated representative in Washington’s state legislature (D, WA), and Joel Castón, who was elected as an advisory neighborhood commissioner in DC while incarcerated, being the first person to do so in DC [48,50,51]. Especially considering the dampened rates of civic participation for those who have been system-involved [52,53], utilizing narratives designed to increase empathy – in this case, for individuals with criminal records – could also drive support and action for decreasing barriers to system-involved individuals’ voting access, providing an opportunity to (potentially) expand the voter base. Though some evidence suggests that experiencing incarceration may serve to shift ideologies further to the right [54], whether that would remain true outside of status quo political messaging (i.e., operationalizing stigmatizing labels of individuals with records) remains unclear.

Thus, this study explores what may have been the impact of propagating such labels on Democrats’ perceptions of people with criminal records, and to what extent does the propagation of such stigmatizing terminology impact how well Democrats perceive to be represented by their own elected officials? Additionally, this study seeks to understand whether providing a counterexample to Trump – highlighting the *perspective* of a formerly system-involved representative on the propagation of this label by the Democratic party – may counter-act potential negative impacts of the Party’s Trump advertisement. To test these questions, this study utilized a survey experimental design to test the following effects amongst self-identified Democrats in the U.S.: a) the impact of exposure to an advertisement of Trump with a stigmatizing label of “convicted criminal,” relative to no advertisement; and b) the impact of reframing that advertisement by juxtaposing it with a preliminary counter-narrative, exposing the perspective of a formerly system-involved leader and their take on the framing of Trump as a ‘criminal,’ relative to just the advertisement alone.

## Materials and methods

### Study purpose and survey design

This study was approved by the Colorado State University Institutional Review Board (IRB), Protocol #7059. All participants in this study provided informed consent for participation. Specifically, the unsigned consent form was provided at the beginning of the study, and participants were instructed that they could provide their informed consent by clicking forward in the survey. This was approved by the IRB (i.e., the protocol included an approved waiver of documentation of consent). There were two purposes of this study. The first of these was to test the impact of exposure to the Trump advertisement on Democrats’ perceptions of individuals with criminal records, as well as their perceptions of elected officials’ motivations (i.e., the extent to which they are motivated by serving the public, as opposed to their own self-interest.) However, the primary purpose of this study was to test whether providing a counter-narrative prior to being presented with the Trump advertisement significantly impacted how Democrats responded to the advertisement. Specifically, this counter-narrative utilized perspective-getting, sharing the experience of a state Assemblymember who was previously incarcerated (i.e., has a criminal record,) and is dedicated to using their platform to serve the public and advocate for system-impacted populations being represented in government.

**Fig 1:**
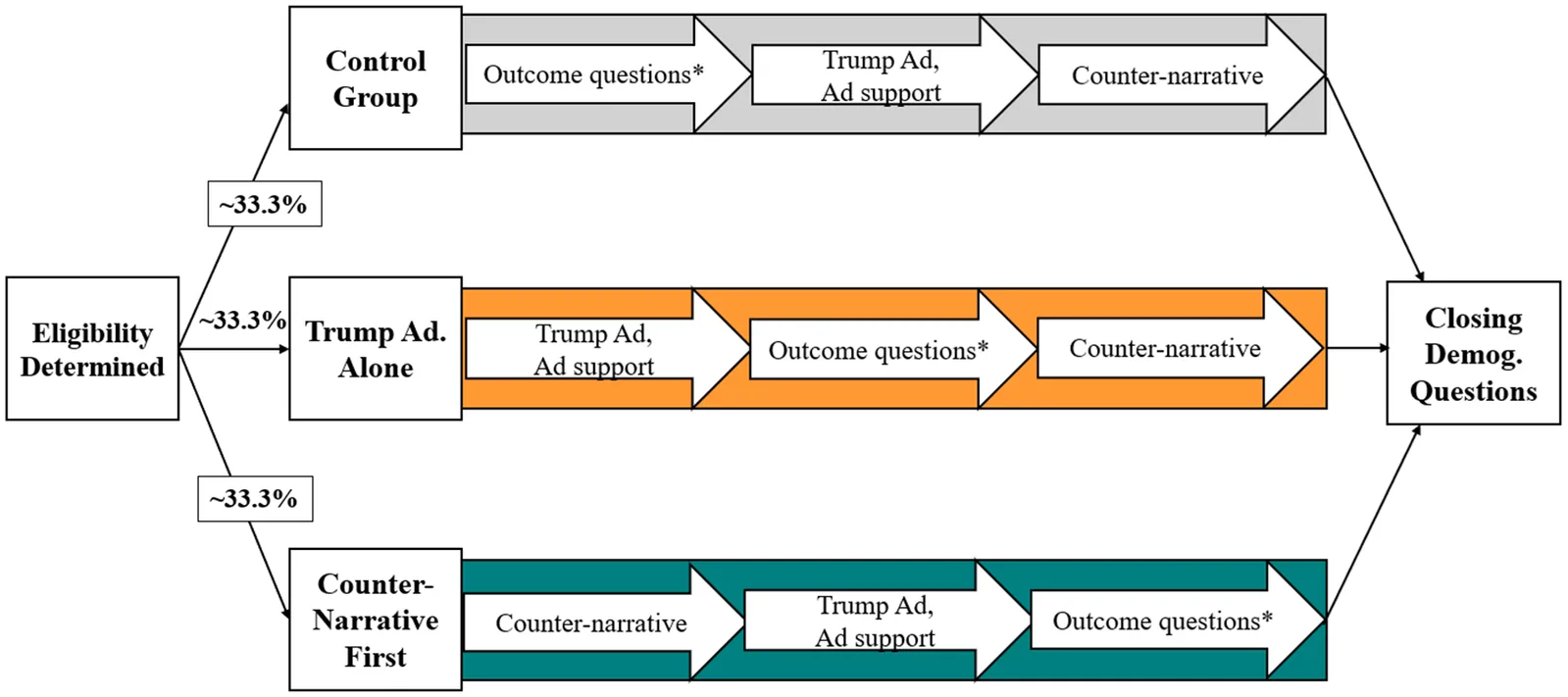
Flowchart of survey by treatment arm. The outcome questions arrow includes all questions that are utilized as outcomes in this study, except for the advertisement support question, which is asked immediately after being presented with the advertisement itself. The “control group” is specifically those who were assigned to answer all outcome questions first (with exception of the advertisement support question,) while “Trump Advertisement (Ad.) Alone” were those who were assigned to be presented with the Trump advertisement first. Finally, the “counter-narrative first group” receives the advertisement only after being presented with a perspective-getting counter-narrative.

Thus, the 2,439 participants were allocated approximately evenly across one of three different treatment groups: a) the *Control* group (*n* = 813); b) the *Trump Ad Alone* group (*n* = 814); and c) the *Counter-Narrative First* group (*n* = 812). The first of these was the *Control* group, which presented all outcome questions first, followed by the Trump advertisement stimulus (and associated questions related to the advertisement), followed by the counter-narrative. The *Trump Ad Alone* group presented participants with the advertisement (and its associated questions) first, followed by the remaining outcome questions, and subsequently the counter-narrative. Finally, the *Counter-Narrative First* group presented the Trump advertisement (and its associated questions) only after being shown the counter-narrative. Then the outcomes were presented. Sociodemographic questions closed out the survey for all treatment arms. Fig 1 presents the flowchart of this survey process for ease of interpretation.

Regardless of when participants were presented with the Trump advertisement, the content was (importantly) constant across treatment arms. Participants were presented with the advertisement, including a description of its use by the Democratic party in 2024 presidential election. Below this descriptive text, participants were presented with one of the images utilized in advertisements within the 2024 presidential election, specifically President Donald J. Trump’s mugshot, which was labeled, in all capital letters, “CONVICTED CRIMINAL.” Due to not possessing the copyright for this image, it has not been included within the manuscript. Participants were immediately presented three questions about the advertisement on the same page (see the Outcomes section below for greater details).

The counter-narrative, on the other hand, provided no visual stimulus, but rather an excerpt for participants to read. This excerpt was from the article entitled, “Could a formerly incarcerated lawmaker reshape justice policy in the NV Legislature?,” by Michael Lyle, published in the *Nevada Current* on November 5, 2024 [55]. The text was lightly edited for brevity, to remove mention of the state in which this individual is an elected official, and their first name, to more narrowly define the counter-narrative treatment. Importantly, this excerpt included both perspective-getting, where Jackson shared their feelings on the election in their own words, and specifically highlighted the importance of redemption, which has been demonstrated in prior literature to be a key predictor of positive perceptions of individuals with records [56–58]. The edited excerpt from Lyle’s 2024 article read as follows:

*“Shortly after his release from being incarcerated, Jackson knew he wanted to give back and help other people impacted by the criminal justice system. Every election cycle he knocked on doors for candidates who backed criminal justice reforms, and every legislative session he would lobby for what he believes are smarter policies that affect people impacted by the justice system. ‘There is absolutely a need for local, state, federal representation of formerly incarcerated,’ Jackson said. ‘I felt like I had to become an advocate for that population.’*

*[During the 2024 presidential election], Democrats quickly sought to frame the race between Harris and Trump as ‘the prosecutor versus the felon.’ Jackson found the contrast hurtful and undercut the work he had done since being incarcerated. He said there is a clear difference between a formerly incarcerated person who sought rehabilitation, served their time and was released, and Trump. Jackson said ‘he’s not trying to take accountability for his mistakes...’*

*As the state considers what justice reforms to pursue and how those laws are written, Jackson said he intends to provide representation for those impacted by the justice system... But this time, it would be as a lawmaker. ‘I think having that mistake showed me that anyone could make a mistake,’ he said.”*

### Hypotheses

All data and code, as well as the pre-registration and pre-analysis plan for this study are available via OSF. These data are available on the published and public OSF project page for this work, “Representation, Political Advertisement, & The Lens of System Involvement,” (https://osf.io/zq8dh/overview). There were two sets pre-registered hypotheses for this study, where the first (Hypothesis Set 1) corresponds with testing the impact of exposure to perspective-getting counter-narrative immediately before exposure to the Trump ad, relative to only being presented with the Trump ad. The second set (Hypothesis Set 2) includes tests of the advertisement’s impact, relative to no exposure to the advertisement prior to answering outcome questions. The pre-registered hypotheses in Set 1 were as follows:

Hypothesis 1A: Exposure to the counter-narrative first, describing a public service-oriented individual who has a criminal record, will ***significantly decrease support for the use of the advertisement***, on average, relative to exposure to the advertisement of Trump being labeled as a “convicted criminal” alone.

Hypothesis 1B: Exposure to the counter-narrative first, describing a public service-oriented individual who has a criminal record, will ***significantly increase (positive) perceptions of individuals with criminal records***, on average, relative to exposure to the advertisement of Trump being labeled as a “convicted criminal” alone.

Hypothesis 1C: Exposure to the counter-narrative first, describing a public service-oriented individual who has a criminal record, ***will significantly increase the perceived value of representation in government***, on average, relative to exposure to the advertisement of Trump being labeled as a “convicted criminal” alone.

Hypothesis 1D: Exposure to the counter-narrative first, describing a public service-oriented individual who has a criminal record, will ***significantly decrease the perceived quality of Democratic representation in federal government***, on average, relative to exposure to the advertisement of Trump being labeled as a “convicted criminal” alone.

Hypothesis 1E: Exposure to the counter-narrative first, describing a public service-oriented individual who has a criminal record, will ***significantly increase the perceived complexity of representation in government***, on average, relative to exposure to the advertisement of Trump being labeled as a “convicted criminal” alone.

Hypothesis 1F: Exposure to the counter-narrative first, describing a public service-oriented individual who has a criminal record, will ***significantly increase perceived (positive) motives of elected officials*** (i.e., more strongly believe that they are motivated by serving the public, versus serving self-interest), on average, relative to exposure to the advertisement of Trump being labeled as a “convicted criminal” alone.

The second set of pre-registered hypotheses were as follows:

Hypothesis 2A: Being presented with the Democratic party’s Trump advertisement alone, labeling him as a “convicted criminal,” will ***significantly decrease (positive) perceptions of individuals with criminal records***, on average, relative to not being exposed to this advertisement

Hypothesis 2B: Being presented with the Democratic party’s Trump advertisement alone, labeling him as a “convicted criminal,” will ***significantly decrease (positive) motives of elected officials*** (i.e., more strongly believe that they are motivated by self-interest over serving the public), on average, relative to not being exposed to this advertisement.

As specified in the pre-registered, pre-analysis plan, supplemental analyses will also explore potential heterogeneity by system-impact status (i.e., whether someone identifies as having previously been involved in the system or impacted by the system through their loved one’s *involvement*.)

### Outcomes

The primary outcomes of interest in this study were: a) perceptions of individuals with criminal records; b) perceived value of representation; c) perceived quality of Democratic representation in federal government; d) complexity of representation; e) motives of elected officials; and f) advertisement support. The perceived value of representation and complexity of representation outcomes are the only of which are measured via individual questions. Both questions asked participants to state the extent to which they agreed with each statement presented on a seven-point Likert scale, ranging from “Strongly disagree” to “Strongly agree.” The perceived value of representation outcome was measured by agreement to the statement, “Having representatives with a wide array of life experiences is crucial to strengthening our democracy,” while the complexity of representation question was measured by agreement to the statement, “Having representatives with a wide array of life experiences is really only beneficial when our representatives effectively advocate for the needs of their constituents.”

The remaining four outcomes are measured as indices, averaged over each question included in the index, and subsequently standardized for ease of interpretation. The perceptions of individuals with criminal records outcome included an index, where participants were presented with five statements and were asked how much they agreed with each on a seven-point Likert scale from “Strongly disagree” to “Strongly agree.” Example statements in this index included “People with criminal records deserve to have the *same level of opportunity* for civic participation as those without criminal records,” and “Most people with criminal records do not positively contribute to their communities,” the latter of which was reverse-coded. In terms of reliability, the Cronbach’s alpha coefficient was 0.71, reflecting the lower end of the threshold for adequate reliability [59]. Thus, supplemental analyses will explore effects on individual items in the index.

The perceived quality of Democratic representation in government index included three items, measured on a seven-point Likert scale from “Strongly disagree” to “Strongly agree,” including statements such as “Democrats in federal government are respectful and tolerant of different types of people.” There was strong reliability for this index, with a Cronbach’s alpha coefficient of 0.90. The motives index included four questions, asking participants the extent to which they felt that elected officials were motivated to serve the public, as opposed to their own self-interest. These measures were adapted from those utilized by the Pew Research Center. Each item was measured on a seven-point Likert scale, where a value of ‘1’ represented someone completely serving their own interests, ‘4’ represented equally serving self-interest and the public, and ‘7’ represented these officials being completely motivated to serve the public. The four questions were specific to all party members in either state or federal government and Democrats in state or federal government. The motives index has sufficient reliability with a Cronbach’s alpha coefficient of 0.90.

Finally, the advertisement support index included two questions on the extent to which participants supported the use of the advertisement labeling Trump as a “convicted criminal.” The two items were again measured the same seven-point Likert agreement scale and included: a) “This is an effective advertisement;” and b) “I support use of this advertisement.” The advertisement support index has sufficient reliability with a Cronbach’s alpha coefficient of 0.80. Supplementary analyses will also explore effects on the third advertisement-related outcome that was intended to be left out of the advertisement support question but aimed to be exploratorily analyzed on the extent to which participants felt that most Democrats supported the use of the advertisement. Lastly, a manipulation check was included after exposure to the narrative, specifically asking participants to state the extent to which they agreed with the statement, “I had an emotional reaction to this narrative.”

### Data

The survey was fielded through Prolific, an online survey platform that is commonly utilized for conducting survey-based research, which has been demonstrated as a reliable platform for obtaining high-quality data. 2,500 participants were recruited for this study, between July 12^th^ and 13^th^ of 2025 [60]. This survey was fielded amongst individuals residing within the United States who spoke fluent English and were members of the Democratic Party. The latter inclusion criteria is important, given the purpose of the study is to understand the impacts of Democratic Party advertising on feelings of being adequately represented and related outcomes, thus the study sample focused only on their constituents. While 2,500 participants completed the survey, there were 2,729 total attempts, which includes those who did not pass the initial attention check or needed to request a return of their submission without penalty. The latter of which includes those who answered only their Prolific ID, the attention check, and a question about their political affiliation, and reported that they did not identify as a Democrat. Of the 2,729 total attempts, 2,481 were retained after dropping those who failed the attention check, were duplicate observations based on stored Prolific identifier embedded data, or provided the wrong completion code (e.g., those who were asked to request a return given their political affiliation provided on the survey did not match what was provided to Prolific,) or no code. After this step, responses that were likely to be bots were also removed. Specifically, to accomplish this, the Qualtrics reCATPCHA score was utilized, which is built upon Google’s implementation of reCAPTCHA, embedded within the survey but not visible to participants. Specifically, these scores range from zero to one, where values above 0.50 are purported to be likely human responses [61]. Of course, there are valid concerns with utilizing this threshold, especially with the rise of broadly-accessible generative AI agents and empirical evidence documenting the potential for large-scale threats to survey-based research – all of which will be discussed in greater detail in the limitations [62–65]. However, emerging literature highlights that Prolific, relative to other survey platforms, tends to have far less prevalent use of generative AI within responses (around 6%), relative to other platforms, such as MTurk (around 41%) [66]. Approximately 1.69% of responses post-removal of duplicates, incorrect/no codes, and failed attention checks were flagged as not being a likely human response, and were also dropped. No observations were flagged as coming from the same IP addressing, leaving the remaining, final analytical sample size of 2,439 participants.

## Results

Overall, there were 2,439 participants as part of the final sample, a majority of which (59.4%) identified as women, with 36.8% identifying as men, and 3.8% identifying as non-binary and/or another gender identity. Most participants were White (70.6%), with 16.4% identifying as Black or African American, 8.9% as Asian or Asian American, 8.3% as Latinx, and 1% identifying as Native Hawaiian, other Pacific Islander, another race and/or ethnicity, or preferred not to state their race. Most participants had a four-year degree or higher (60.8%) and 21.4% of the sample identified as being system-impacted. The average age of participants was 43.4 years old.

Given that the methodology utilized was a survey experiment, verifying that there is no evidence of failed randomization is a crucial assumption to check. Table 1 below presents the balance table, reporting proportions of sociodemographic variables across the study sample by treatment group assignment (i.e., the *Control* group – “C” in Table 2 – the *Trump Ad Alone* group – “T” in Table 2 – and the *Counter-Narrative First* group – “N” in Table 2) Tests for potential imbalance (i.e., significant differences in sociodemographic factors by treatment group assignment) were performed with least squares regression, utilizing heteroskedasticity-consistent standard error estimator, HC-2, with the treatment indicators alone (i.e., $D_{i\text{0}}$ and $D_{i\text{2}}$ where $D_{i\text{0}}=\text{1}$ when $T_{i}=\text{0}$, or assignment to the *Control* group and 0 otherwise, and $D_{i\text{2}}=\text{1}$ when $T_{i}=\text{2}$, or assignment to the *Counter-Narrative First* group and 0 otherwise). There were no statistically significant differences between either the *Control* and *Trump Ad Alone* groups, nor the *Trump Ad Alone* and *Counter-Narrative First* group. This means that there is no statistical evidence of imbalance between treatment arms that are being compared to one another in this study. However, given that there are smaller, relative differences across treatment arms, the primary models for this analysis include both the treatment indicator and a set of covariates, all of which are noted below and utilized as specified in the pre-registration. For transparency, reported models will report both the treatment-indicator only models and primary models with covariates.

**Table 1:** Balance table.

Variable	Level	Narrative (N)	Trump (T)	Control (C)	Sig (C/T)	Sig (N/T)
Gender	Man	0.371	0.362	0.37	0.743	0.729
	Woman	0.587	0.602	0.592	0.671	0.551
	Non-Binary	0.027	0.018	0.015	0.563	0.242
	Other Gender	0.015	0.017	0.023	0.378	0.697
Race and/or Ethnicity	American Indian or Alaskan Native	0.018	0.015	0.021	0.347	0.556
	Asian	0.087	0.082	0.097	0.295	0.711
	Black	0.164	0.17	0.159	0.554	0.756
	Latinx	0.075	0.079	0.095	0.249	0.791
	Native Hawaiian, Pacific Islander or PNTS	0.009	0.014	0.009	0.345	0.346
	White	0.708	0.713	0.696	0.47	0.845
Education Level	Less than four-year degree	0.378	0.392	0.405	0.599	0.567
System-Impacted	System-Impacted	0.209	0.219	0.213	0.773	0.647
Age	Age	43.697 (14.56)	43.445 (14.028)	42.983 (14.14)	0.508	0.722
Racial Attitudes	Racial Attitudes	0.010 (0.979)	0.038 (0.985)	−0.049 (1.034)	0.082	0.565

*Note: Narrative (N), Trump (T), and Control (C) columns all reflect the proportion of that specific treatment arm that identify as that given identity or, when reporting on numeric sociodemographic variables, the mean and standard deviation within that group (i.e., the *Counter-Narrative First, Trump Ad Alone,* and *Control* groups, respectively.) The Sig (C/T) column reports the significance level of the regression with each sociodemographic variable as the outcome and the coefficient on the comparison between the *Control* group and the *Trump Ad Alone* group (the latter of which is the reference category.) From that same regression, we can also report the significance of the *Counter-Narrative First* group, relative to the reference category of *Trump Ad Alone* on each sociodemographic variable, which is reported in the Sig (N/T) column. Note that gender and race/ethnicity were asked in such a manner that allowed participants to select all identities that applied to them.

**Table 2:** Model Results for hypothesis set 1 (effect of counter-narrative, relative to trump advertisement alone).

	Ad. Support		Perceptions of Those with Records		Value of Rep.		Quality of Democratic Rep.		Complexity of Rep.		Public-Serving Motives	
	*T* only	*T* + *X*	*T* only	*T* + *X*	*T* only	*T* + *X*	*T* only	*T* + *X*	*T* only	*T* + *X*	*T* only	*T* + *X*
Coefficients	−0.18	−0.172	0.830	0.833	0.785	0.790	−0.339	−0.332	−0.065	−0.062	−0.102	−0.098
Robust S.E.	0.049	0.048	0.042	0.041	0.048	0.047	0.049	0.048	0.049	0.048	0.050	0.048
*p*-value	< 0.001	< 0.001	< 0.001	< 0.001	< 0.001	< 0.001	< 0.001	< 0.001	0.1816	0.1987	0.0412	0.0431
*N*	2,439		2,439		2,439		2,439		2,439		2,439	

*Note: Models reported include the “T only” models (i.e., treatment indicator only) and the “*T* + ***X***” models, which include the following covariates, as specified in the pre-registration and pre-analysis plan: a) gender; b) race and/or ethnicity; c) education (less than four year degree indicator); d) whether someone identifies as being system-impacted or not; e) age; and f) the pre-treatment racial attitudes index. Of the above significant outcomes, only the public-serving motives outcome would not be significant after multiple comparisons corrections, particularly Bonferroni.

Note that comparisons on balance between the *Control* group and the *Counter-Narrative First* group are not included in Table 1, as none of the hypotheses test for differences between these two groups specifically. Given that the design employed was *not* a full factorial (i.e., there was no way to isolate the specific effect of the counter-narrative *alone*, given the design,) direct comparisons between these two groups were not prioritized in hypothesis testing. Note that this means that differences between this group are *not* the effect of the counter-narrative, which was paired with (immediately followed by) the Trump ad in the *Counter-Narrative First* treatment arm. These design considerations will be discussed further in the Discussion section.

### Main results

First, analysis of statistical differences on the manipulation check demonstrates the expected effect of the counter-narrative. Specifically, those assigned to the counter-narrative first condition reported significantly higher, on average, agreement to having an emotional response, relative to both the *Control* group (ATE = −0.156, robust SE = 0.047) and the *Trump Ad Alone* (ATE = −0.135, robust SE = 0.048), while these latter two groups were not significantly different from one another (difference of 0.021 and robust SE of 0.05). Thus, priming participants with the counter-narrative had the intended effect. This is also useful in providing some evidence against the potential that any subsequent results are largely driven by social desirability bias: if that were true, the emotional reactions to the narrative in the control group would have likely been driven by exposure to the outcome questions that they had answered, which does not appear to have been the case.

Tables 2 and 3 report the results of hypothesis testing for Hypothesis Set 1 and 2, respectively. For all hypotheses, the primary estimate of interest is the average treatment effect (ATE), representing the mean difference in outcomes between two treatment arms. To estimate each ATE, least squares regressions with heteroskedasticity-consistent standard error estimators, HC-2, were utilized. Both the treatment indicator only and the primary models (which include covariates, as pre-specified), are reported for transparency. Each of the models in this analysis utilize the full study sample and set the reference category in the regression for the treatment indicator to be those assigned to the *Trump Ad Alone* group (i.e., $T_{i}=\text{1}$, $D_{i\text{0}}=\text{0}$, and $D_{i\text{2}}=\text{0}$). The model equation is as follows:

**Table 3:** Model results for hypothesis set 2 (effect of trump advertisement, relative to control group).

	Perceptions of Those with Records**		Value of Rep.		Quality of Democratic Rep.		Complexity of Rep.		Public-Serving Motives**	
	*T* only	*T* + *X*	*T* only	*T* + *X*	*T* only	*T* + *X*	*T* only	*T* + *X*	*T* only	*T* + *X*
Coefficients	−0.819	−0.835	−0.773	−0.791	0.308	0.289	0.064	0.056	0.147	0.130
Robust S.E.	0.044	0.044	0.049	0.048	0.049	0.048	0.050	0.050	0.051	0.049
*p*-value	< 0.001	< 0.001	< 0.001	< 0.001	< 0.001	< 0.001	0.2016	0.2568	0.0037	0.0081
*N*	2,439		2,439		2,439		2,439		2,439	

*Note: The only pre-registered, apriori-selected outcomes that were hypothesized to be impacted by Trump advertisement were the following two indexes, which are indicated by “**” in the above table: a) perceptions of those criminal records; and b) motives of representatives in federal and state government (i.e., the extent to which all party members or Democrats in federal or state government are motivated to serve the public, rather than their own self-interest.) The remaining outcomes are provided for the sake of transparency. The advertisement support outcome is not included above as this is asked to all groups, but only after exposure to the advertisement (i.e., the control group received the advertisement and narrative only after answering all other outcome questions.) Models reported include the “T only” models (i.e., treatment indicator only) and the “*T* + ***X***” models, which include the following covariates, as specified in the pre-registration and pre-analysis plan: a) gender; b) race and/or ethnicity; c) education (less than four year degree indicator); d) whether someone identifies as being system-impacted or not; e) age; and f) the pre-treatment racial attitudes index. Of the above significant outcomes, only the public-serving motives outcome would not be significant after multiple comparisons corrections, particularly Bonferroni.


$$\begin{array}{c} Y_{i}=b_{\text{0}}+\beta_{\text{0}}D_{i\text{0}}+\beta_{\text{2}}D_{i\text{2}}+\gamma X_{i}+\varepsilon_{i} \end{array}$$

Where tests of the effect of exposure to the Trump advertisement (*Trump Ad Alone* – *Control*) are recovered by $\beta_{\text{0}}$ (after multiplying coefficients by −1) and tests of the impact of the counter-narrative, relative to the Trump ad alone, are recovered by $\beta_{\text{2}}$.

First, beginning with Hypothesis Set 1 (H1), the purpose was to test the impact of exposure to the counter-narrative prior to the Trump advertisement, relative to only being presented with the Trump advertisement. In other words, these models estimate the ATE, reflecting adjusted mean differences in the average outcomes in the *Counter-Narrative* group and the *Trump Ad Alone* group. All pre-registered, apriori hypotheses were supported, with exception of one (H1E); however, it is also important to note that the significance of H1F’s test would no longer hold after application of a (Bonferroni) multiple comparisons correction.

Starting with H1A, exposure to the counter-narrative significantly decreased support for the Trump advertisement, relative to the Trump advertisement alone (${\hat{\beta }}_{\text{2}}$ = −0.172 SE = 0.048). Given this was a two-item index, covering both explicit support and perceived effectiveness of the advertisement, additional analyses included testing the effective of the counter-narrative relative to the Trump ad alone on each individual item in the index. Both coefficients are negative, including that of the item “This is an effective advertisement” and “I support the use of this advertisement”, though only the explicit support for the advertisement item was statistically significant. The most profound impact of the counter-narrative, unsurprisingly, given the content of the counter-narrative, was on the perceptions of individuals with criminal records (H1B): exposure to the counter-narrative prior to being presented with the Trump advertisement increased positive perceptions of individuals with criminal records by more than 0.8 standard deviation (SD) units, compared to those presented with the Trump advertisement alone (${\hat{\beta }}_{\text{2}}$ = 0.83, SE = 0.041). H1C’s tests reported similar magnitudes of effect, with the initial exposure to the counter-narrative demonstrating a 0.79 SD unit increase in the perceived value of diversity of life experience as an important component of strengthening democracy, relative to those only presented with the Trump advertisement before answering outcome questions (${\hat{\beta }}_{\text{2}}$ = 0.790, SE = 0.047). Additionally, evidence supported H1D, namely that exposure to the counter-narrative significantly decreased quality of Democratic representation in federal government, relative to just Trump advertisement exposure (${\hat{\beta }}_{\text{2}}$ = −0.332, SE = 0.048). Finally, there was evidence in support of H1F – though importantly, this finding would no longer be significant after applying a Bonferroni multiple comparisons correction. Specifically, exposure to the counter-narrative significantly (at the α = 0.05 threshold *without* a multiple comparisons correction), reduced the extent to which participants believed that elected officials’ motivations were to serve the public, versus serving their own self-interest (${\hat{\beta }}_{\text{2}}$ = −0.098, SE = 0.048). H1E was unsupported, namely there were no significant differences between the *Counter-Narrative First* and *Trump Ad Alone* groups on the outcome related to the complexity of representation (${\hat{\beta }}_{\text{2}}$ = −0.062, SE = 0.048).

Hypothesis Set 2 (H2) has only two hypotheses, though results for all outcomes are presented for the sake of transparency, with exception of the advertisement support outcome. The advertisement support outcome is *not included* in Table 3 as this question is asked to all treatment arms only after exposure to the advertisement. Thus, the control group was exposed to the advertisement and the counter-narrative, but only after answering all other outcome questions. Both H2A and H2B were supported, namely, exposure to the Trump advertisement alone significantly *decreased* positive perceptions of individuals with criminal records (${\hat{\beta }}_{\text{0}}$ = −0.835, SE = 0.044) and significantly *increased* participants beliefs that elected officials’ motivations were to serve the public, versus serving their own self-interest (${\hat{\beta }}_{\text{0}}$ = 0.130, SE = 0.049). Notably, both beliefs that diversity of individuals’ life experiences as crucial to democracy and perceived quality of Democratic representation were also significantly impacted by the Trump advertisement, which were not hypothesized.

### Supplemental analyses

Supplemental analyses are provided to help clarify findings and explore results that were identified as secondary analyses. Thus, while this means findings in this section should be interpreted with caution, these results are presented to provide transparency and aid interpretation of this study’s findings. In order, this section will describe: a) treatment effects on individual items for the perceptions of individuals with criminal records index, given its marginally acceptable reliability coefficient; b) summarization of the sociodemographic factors that were significantly predictive of the outcomes of interest; and c) heterogeneity in results by system-impact status.

**Fig 2:**
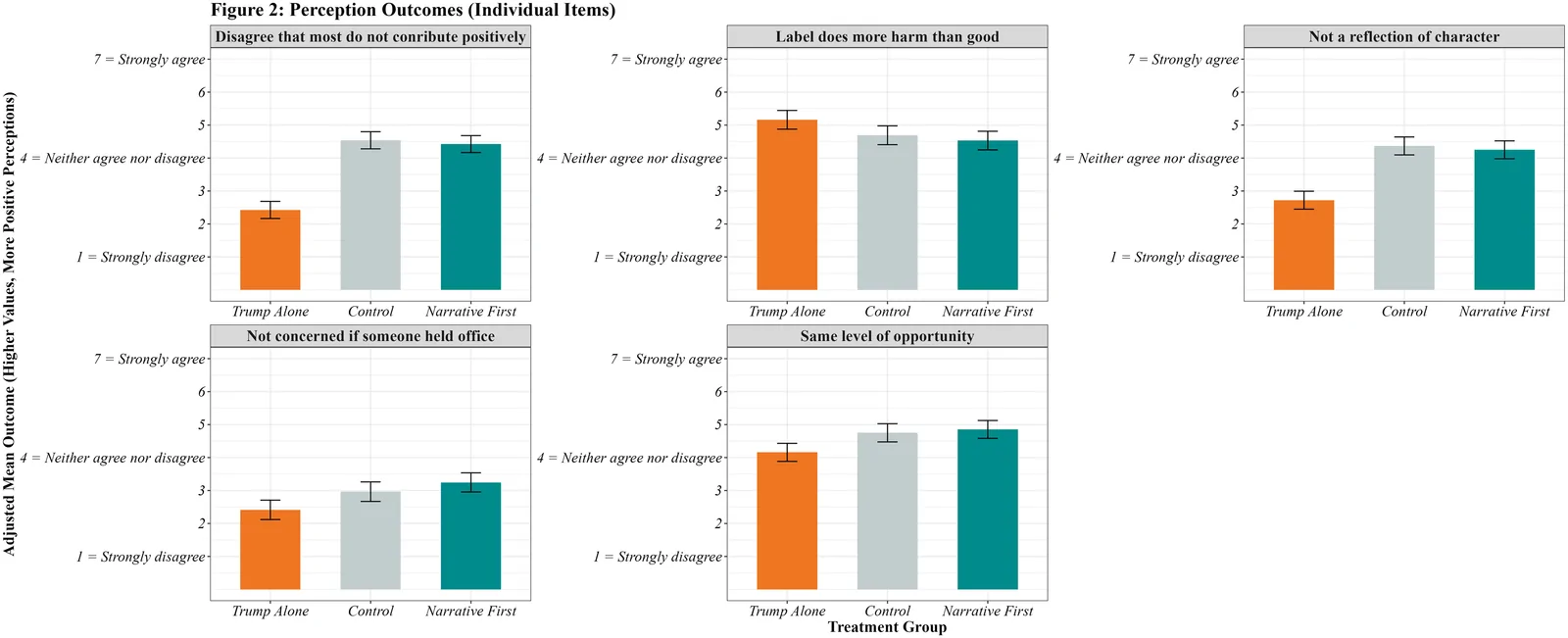
Adjusted means for perceptions of individuals with criminal records. The outcome abbreviations are detailed here: Disagree that most do not contribute positively = “Most people with criminal records do not positively contribute to their communities” (reverse-coded); Label does more harm than good = “Labeling people with criminal records as ‘criminals’ may do more harm than good”; Not a reflection of character = “Having a criminal record is a strong reflection of someone’s overall character” (reverse-coded); Not concerned if someone held office = “I would be concerned if I lived somewhere in which someone with a criminal record held a position with political power” (reverse-coded); Same level of opportunity = “People with criminal records deserve to have the same level of opportunity for civic participation as those without criminal records.” Comparisons between the *Trump Ad Alone* group with both the *Control* and *Counter-Narrative First* groups were significantly different for all items.3.

First, analyses of the effects of the Trump advertisement (relative to no advertisement exposure) and the counter-narrative prior to the Trump advertisement (relative to the advertisement alone) impacted each *individual* item in the perceptions of individuals with criminal records index largely in line with the primary hypothesis. Estimates of the ATE presented in-text utilize the standard deviation unit scale for each outcome to more clearly signal the magnitude of the effect, though the regression-adjusted means are presented on their original, seven-point Likert scale in Fig 2 below for aiding practical understanding.

Both the *Counter-Narrative First* and the *Control* groups’ model-adjusted averages were significantly different from that of the *Trump Ad Alone* group for each individual item, though one was directionally opposite than what was hypothesized. Again, ${\hat{\beta }}_{\text{0}}$ (multiplied by −1) recovers the ATE of exposure to the Trump advertisement and ${\hat{\beta }}_{\text{2}}$ represents the ATE of exposure to the counter-narrative prior to the ad, relative to the advertisement alone. Items that had demonstrated effects in the hypothesized direction were, framed with reverse-coding where applicable: a) people with criminal records deserve to have the same level of opportunity for civic participation (${\hat{\beta }}_{\text{0}}$ = −0.386, SE = 0.049) and (${\hat{\beta }}_{\text{2}}$ = 0.453, SE = 0.048); b) not being concerned if they lived somewhere where a person with a criminal record held a position with political power (${\hat{\beta }}_{\text{0}}$ = −0.355, SE = 0.048) and (${\hat{\beta }}_{\text{2}}$ = 0.536, SE = 0.046); c) having criminal records is not a strong reflection of someone’s overall character (${\hat{\beta }}_{\text{0}}$ = −1.018, SE = 0.043) and (${\hat{\beta }}_{\text{2}}$ = 0.945, SE = 0.043); and d) disagreeing that most people with criminal records do not contribute positively to their communities (${\hat{\beta }}_{\text{0}}$ = −1.251, SE = 0.041) and (${\hat{\beta }}_{\text{2}}$ = 1.183, SE = 0.040). The outcome that was impacted in the direction opposite of what was expected was that labeling people with criminal records as criminals may do more harm than good (${\hat{\beta }}_{\text{0}}$ = 0.328, SE = 0.048) and (${\hat{\beta }}_{\text{2}}$ = −0.441, SE = 0.047). It could be that the question was misinterpreted, that solely providing the Trump advertisement as a stimulus reduces the extent to which participants embraced nuance, and/or that this is item measures something more distinct from perceptions of individuals with records.

Regarding the most predictive factors related to outcomes of interest, outside of the treatment effects themselves, Table 4 below displays all factors that were significant for at least one of the six outcomes in this study. Note that Appendix B includes the full tables, with the inclusion of the full categories for education. Some significant variation was observed between gender identities, as well as racial and ethnic identity (i.e., both of these questions were a select all that apply.) Largely, these factors were most predictive of representation-focused outcomes, including the quality of representation and the extent to which elected officials are public service-minded. System-impacted status also contributed significant variation, such that those that identified as being system-impacted held more positive perceptions of individuals with records, relative to those who did not disclose system-impact status, on average. Most strongly and consistently predictive of nearly all outcomes, with exception of the public service motivation of elected officials, was this sample of Democrats’ racial attitudes. Namely, more positive racial attitudes were associated with stronger support for the advertisement, more positive perceptions of individuals with criminal records, more perceived value of diversity of life experience in representation, better quality of representation, and greater agreement that symbolic representation may not be sufficient.

**Table 4:** Significant predictors of outcome variables.

Outcome/ Factor	Ad. Support	Perceptions of Those with Records	Value of Represent.	Quality of Represent.	Complexity of Represent.	Public-Service Motives
Man+				**		**
Woman+				***		***
Asian		**		*		*
Black		***		*		**
Hispanic, Latinx	*	**				
Native Hawaiian or Other Pacific Islander+						**
Some college						
2-year degree						
4-year degree						
Master’s degree or higher	**					
Age (in years)			**	***		***
System-impacted		***				
Racial attitudes (index)	***	***	***	**	***	

Note: Significance in the above table is denoted as follows: * *p* < 0.05; ** *p* < 0.01, *** *p* < 0.001.

Finally, in regard to the heterogeneity of treatment by whether someone identified themselves as being system-impacted (*n* = 521) or not (*n* = 1,918), focusing specifically on the primary outcomes of interest, there was evidence of significant heterogeneity in the effect of the *Trump Ad Alone* relative to the *Control* group for perceptions of individuals with records, and for the effect of the *Counter-Narrative First*, relative to the *Trump Ad Alone* group, for both the perceptions of individuals with records and the complexity of representation outcome.

**Fig 3:**
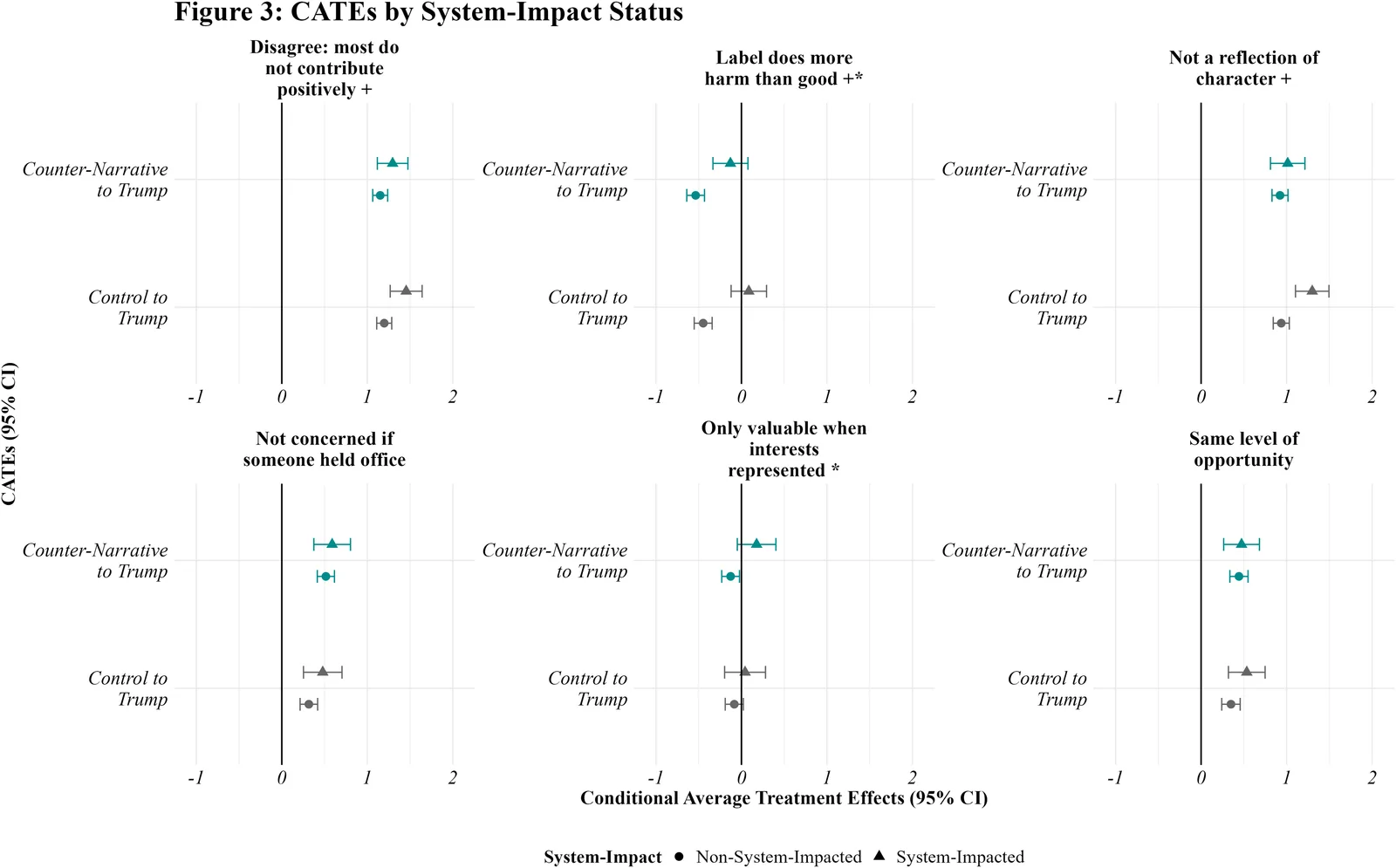
Conditional average treatment effects by system-impacted status. This figure displays conditional average treatment effects (CATEs) by system-impact status, where non-system-impacted means they were not system-impacted or preferred not to state their system-impacted status. *Y*-axis categories represent the comparison of interest (i.e., either *Counter-Narrative* to *Trump Ad Alone* or the *Control* compared with *Trump Ad Alone*.) CATEs by system-impact are denoted by shape as presented in the legend. While CATEs are estimated models conditional on system-impacted status, significance from the interaction models is reported as either (or both) “+” for significance on the interaction between system-impacted status and the *Control* group indicator and “*” for significance on the interaction between system-impacted status and the *Counter-Narrative First* indicator. Outcomes are specific to perceptions of individuals with criminal records, given significance of the interaction terms from models that tested treatment effect heterogeneity by system-impact status (and in line with the pre-registered pre-analysis plan.).

Given the mediocre reliability for the perceptions of individuals with criminal records index, results will be presented by individual items for this outcome instead. While the significance of the interaction term in the full model (i.e., treatment indicator terms and their interactions with the indicator for a participant identifying as being system-impacted) determined whether there was significant heterogeneity, for ease of interpretation, conditional average treatment effects (CATEs) presented in Fig 3 are from models estimated on each sub-sample. Fig 3 includes a total of six outcomes – the five items from the perceptions of individuals with records index and the complexity of representation outcome.

In terms of relative magnitude, the effects were greater for those who identified as being system-impacted, compared to those who did not (or preferred not to state their system-impact status). Note that the percentage of individuals in the overall sample that indicated prefer not to state their system-impact status was only 2.8%. Specifically, for the items about possession of having a criminal record being a reflection of someone’s character, that most people with criminal records do not positively contribute to their communities (which was reverse-coded), and that labeling people with records as ‘criminals’ may do more harm than good, the Trump advertisement exposure appeared to be significantly *more* damaging to perceptions of individuals with criminal records for those who are system-impacted. In other words, the coefficient on the interaction term between the *Control* group indicator and system-impacted status was significant and positive. When not reminded of Trump as a benchmark, those who identified as system-impacted reported substantially more positive perceptions of individuals with criminal records, relative to their counterparts who did not disclose being system-impacted. Lastly, the effect of the counter-narrative was heterogeneous by system-impact status, specifically for the outcome of labeling individuals with criminal records as ‘criminals’ doing more harm than good.

## Discussion

Collectively, this study provides evidence as to the extent to which framing Donald J. Trump with stigmatizing labels, such as “convicted criminal” or “felon,” may have had spillover effects on Democrats’ perceptions of individuals with criminal records more broadly, as well as myriad dimensions of perceived representation. Additionally, this work seeks to understand whether providing a counter-stereotype has the potential to counteract negative impacts of propagating such labels. First, there is clear evidence that the Trump advertisement caused decreases in positive perceptions of individuals with criminal records amongst Democrats, but when paired with (and preceded by) a counter-narrative of a dedicated, system-impacted state representative who speaks to the harm of the “criminal” label, these effects were almost entirely ameliorated (${\hat{\beta }}_{\text{2}}$ = 0.833, whereas ${\hat{\beta }}_{\text{0}}$ = −0.835). Thus, while the effect of the Trump advertisement is clear and certainly a cause for concern – which will be further addressed in the Conclusion – the strength of the counter-narrative is not unexpected, but promising, given prior evidence on the effects of perspective-getting and counter-stereotypical narratives on increasing empathy for members of an outgroup [67–70]. The effects of such interventions have also been found to improve attitudes towards system-involved people [46], public sector workers [71], and attitudes towards structural reform, including support for reducing reliance on carceral practices [72].

These results also lend weight to the potential mechanism through which the Democratic Party could have sought to bolster their support, as providing the counter-narrative also worsened perceptions of the quality of Democratic representation, relative to the Trump advertisement alone. Additionally, exposure to the Trump advertisement significantly increased feelings of being well represented by elected officials and the extent to which Democrats perceived that elected officials were motivated by public service over self-interest. It may be that, in relation to Donald J. Trump (i.e., after being exposed to the advertisement,) performance of representatives in federal government is perceived to be comparatively better than the DJT benchmark. Significant differences were also found – counter to hypotheses – for the effect of exposure to the Trump advertisement on valuing diversity in life experiences amongst elected officials. It is possible that exposure to the Trump advertisement encouraged individuals to agree less in the value of diversity of life experiences, given some of those life experiences may include attempting to sway a presidential election for one’s personal benefit and that of the ultra-wealthy through falsification of business records to cover up a scandal (and crucially, from someone showing neither remorse nor accountability). Collectively, these results may simply be Prospect Theory in action: Donald J. Trump may be serving as reference point that may influence Democrats to see other elected officials as comparatively better [73]. Lowering the bar and benchmark against which your representatives are compared is certainly an easier feat than fostering stronger representation and decision-making amongst your party’s representatives.

Additionally, leaning into this counter-narrative as the Party’s rhetoric may have been avoided for myriad reasons, including that the more humanizing approach of highlighting a dedicated formerly system-involved representative may have been considered as ‘too radical.’ This would not be particularly surprising, as recent evidence suggests that more moderate appeals tend to be strategically adopted, such that in primaries, Congressional candidates tend to campaign on more extreme ideals, though may be more likely to temper these messages to appeal more towards the middle when facing well-resourced opponents [74]. While the implications of leaning into stigmatizing rhetoric are concerning, this is not to say that failing to acknowledge or address the criminal conviction of Donald J. Trump would have been advised. While the potential “positive” effects of exposure to the Trump advertisement signal that this may benefit the Democratic party, the magnitude of these effects (approximately 0.30 to 0.34 in absolute value SD units) are considerably smaller than the advertisement’s effects on perceptions of individuals with criminal records (approximately 0.83), and exposure to the counter-narrative significantly decreased support for the advertisement. Still, the effect of the advertisement exposure on perceived quality of representation is non-trivial.

From the supplemental analysis, results suggest that system-impacted individuals may be more apt to understanding the nuance of entanglement in the justice system: on every item related to perceptions of individuals with criminal records, those who identified as being system-impacted reported significantly more positive perceptions overall and within each treatment arm, with only one exception. This is unsurprising, as evidence suggests that personal exposure and belief in possibilities for redemption are associated with having more positive perceptions of individuals with criminal records. While it is possible that the intervention constructs a new category of deservingness, rather than reducing stigma, the empirical relationship between attitudes and personal connection suggests that reduction of stigma is more likely [55–57]. Still, future research should clarify this relationship more deeply. Specifically, for the dimension of whether labeling individuals with criminal records does more harm than good, within the exposure to the Trump advertisement alone group, those who identified as being system-impacted agreed *relatively less strongly*, on average, to this item (mean difference = −0.13, *p* = 0.134), compared to those who did not identify as being system-impacted or did not state their system-impacted status. In other words, the enacted campaign strategy of Democratic Party in the 2024 Presidential Election may have facilitated even greater barriers to solidarity amongst system-impacted constituents, incentivizing the construction deservingness – or lack thereof – of access to positions with political power for individuals with criminal records.

Importantly and unsurprisingly, racial attitudes appeared to be one of the more predictive factors for most outcomes in this analysis, including both types of outcomes related to democratic representation (e.g., quality of representation and valuing diversity of life experiences in representation,) as well as perceptions of individuals with criminal records. On average, more positive racial attitudes were associated with stronger perceptions of representation quality, greater value of the diversity of life experiences in representation, and crucially, more positive perceptions of individuals with criminal records, holding all else equal. This finding lends weight to the analysis, given pre-existing literature highlighting the strong relationships between measures of racial attitudes and political support amongst Democrats, for example, White Democrats’ support for Black politicians [75].

## Conclusion

Collectively, there are several avenues through which future research can improve upon the evidence collected in this study, given its limitations, and to increase the potential for meaningful policy impact. The purpose of this paper was to identify the *potential* for spillover harm that may have been caused in propagating stigmatizing labels (and/or images) on Trump, encouraging further inquiry. As with all research, one study cannot effectively answer all relevant or important research questions, and thus, this work should be considered as preliminary evidence of potential effects of the Democratic Party’s messaging strategy, and a line of work that warrants far more empirical attention than it has received.

Of course, there are limitations to this study, particularly on the dimensions of: a) design constraints and modality of data collection; b) external validity; and c) measurement, all of which can be helpfully addressed and explored in future research. First, the ideal version of this survey experiment would have been to utilize a full factorial design (i.e., including the exposure to the counter-narrative perspective arm alone). This was entirely done a result of binding budget constraints, and the decision on which arm to drop was entirely intentional based on the priority research questions as well as the level of available literature to speak to the potential impacts of the counter-narrative perspective arm alone. Of course, failing to include the counter-narrative only arm limits the ability to interpret the *additional* impact of including it with the advertisements; in other words, the counter-narrative arm cannot be directly compared to the pure control arm to test the impact of the counter-narrative alone because it also involves exposure to the Trump advertisement. This is important, as utilizing a negative campaign advertisement could moderate the effect of the counter-narrative perspective intervention, depending upon one’s personality traits, amongst other potential factors [76]. While this is certainly a considerable limitation, a robust prior literature documents the effects of perspective-getting on perceptions of and empathy toward others, including members of an outgroup, which informed the inclusion of the counter-narrative, given prior literature’s notes of its propensity to significantly shift perceptions [67–70]. Replication of this work with a full factorial design is strongly encouraged, especially since outcomes related to representation quality are far more limited in the perspective-getting literature. Additionally, as the advertisement (i.e., Trump’s mugshot with the words ‘convicted criminal’ imposed on the image) was presented consistently across treatment arms, it is not possible to disentangle the impact of the image from the stigmatizing terminology imposed, and thus, this is recommended for exploration in future work.

It is also incredibly important to acknowledge that this survey was fielded amongst an online panel of survey respondents, and the proliferation of generative AI should cause concerns over the humanity of the responses provided within the survey. While this survey was completed before the introduction of Prolific’s bot detection – which is actively deployed in a beta version at the time this paper was submitted – Prolific has also been documented as having comparatively one of the lowest percentages of bots infiltrating their survey base [66]. Additionally, much of the literature that documents the potential threats and/or prevalence of generative AI in online surveys focuses specifically on open-text threats, with some exceptions (e.g., Westwood, 2025) [62]. Still, the propensity for the standard online survey taker to utilize AI agents to complete close-ended surveys is not well understood. Thus, while concerns for the use of AI in online surveys are valid, these concerns should be considered in line with their context (i.e., open-ended survey responses and in reference to propensity of typical survey participants’ to build and deploy AI agents for completing online surveys with both closed- and open-ended responses).

Relatedly, the extent to which these results would replicate in other contexts and in less-transparent designs is unclear, as is the durability of effects. First, it could be the case that participants were subject to the Hawthorne effect, resulting in outcomes being driven – in some capacity – by social desirability bias. The manipulation check, namely the fact that the *Counter-Narrative First* group exhibited significantly greater emotional reaction to the narrative than either of the other two treatment arms, which were primed with the Trump advertisement *before* the counter-narrative, helps alleviate some concerns but does not rule this out. Thus, future research should employ less transparent designs and data collection via different modalities that can be more impervious to influence from social desirability bias.

Specifically, the single time-point survey experimental approach is a useful first step, but leaves unanswered several key questions related to the ability of these results to replicate: a) for how long do the effects of the counter-narrative prime before the advertisement and the advertisement itself *persist;* b) how may effects vary by single versus repeated exposure to the advertisement and/or the counter-narrative prime?; and b) to what extent would these treatments translate to shifted decision-making and behavior, as opposed to self-reported perceptions and attitudes? While these questions, of course, cannot be concretely answered without future research, prior literature can highlight the potential for the counter-narrative, in particular, to be a promising route for exploration in future study. For example, perspective-getting interventions in field experimental applications across myriad domains, such as support for healthcare for undocumented immigrants, have been found to have quite durable effects, up to four and a half months in prior literature.

Thus, field experimental approaches that can include more concrete measures about behavior and via longitudinal, rather than single time-point designs, should be prioritized. Importantly, *when* such studies are implemented will be important, as this current study was conducted in July of 2025, after the outcome of the election was known and not actively during the campaign. Whether these results replicate during campaign periods is an open question, perhaps especially so for the advertisement’s impacts in particular. Again, an incredibly robust body of literature documents the effects of perspective-getting narratives like the counter-narrative included within this study, on improving empathy for and perceptions of others, including outgroup members, which does not explicitly require proximal timing to specific events in order to be effective [67–70]. Though for understanding advertisement effects in particular, especially as it relates to potential voting behavior, timing of future (field) experimental studies should be in line with election periods.

Finally, future studies should also aim to include a wider array of questions, both related to perceptions of individuals with criminal records and feelings of being represented. Specifically, there are myriad aspects feeling represented (e.g., in terms of policy position, symbolic and active representation on myriad dimensions of identity, etc.), that may be additionally important to explore, and given the reliability statistics for the perceptions of individuals with criminal records index was satisfactory but not necessarily strong, utilizing a more robust set of measures is an important next step. Crucially, racial attitudes were measured as covariates and presented before stimuli; however, understanding the potential for activation of negative stereotypes related to those with criminal records could very likely impact Democrats’ racial attitudes, given the racist stereotypes that are propogated in relation to criminal justice system impact. As such, this should be prioritized as an outcome in future research.

Again, while the effect of exposure to the Trump advertisement in improving perceptions of being well-represented by Democrats was certainly non-trivial, this is not to say that the only viable option for a campaigning strategy was to utilize advertising that has strong potential for negative repercussions for others [8–11]. Instead, a viable, alternate route for political messaging during the 2024 presidential election could have been to acknowledge, but juxtapose, the nature of Trump’s convictions and his broadcasted attitude about his convictions with effective counter-narratives. His responses to the trial were quite salient during this campaign as well, with many articles reporting his comments on the convictions as being the product of Democrats’ efforts to harm his chances at re-election, framing himself as a “political prisoner,” and stating that the trial was “rigged,” and “disgraceful” [1]. Leaning instead into narratives from many devoted, formerly system-involved representatives who seek to give back to and advocate for their communities, could have had greater potential to speak to other formerly system-involved potential voters. Again, future research should be prioritizing field experimental work in election periods to more concretely understand the effects of stigmatizing advertisements – should they continue – or ideally, the effect of such potentially viable alternatives.

This approach also may have been more strategic, given the limited voter turnout amongst individuals with criminal records, particularly those with felony convictions. While empirical findings on the effects of incarceration on voter turnout are tenuous [53,74,75,77,78], there are clear and profound barriers to civic participation, including and beyond voting, for those with records, especially those with felony convictions. In fact, across the U.S. more than 4.6 million individuals are disenfranchised as a result of felony convictions [79]. There is also immense heterogeneity across the U.S. in terms of which (formerly) system-involved individuals are allowed to vote and when. As of November 2025, ten states eliminate the rights of individuals with certain types of convictions from ever voting again, while half of states automatically restore voting rights after incarceration or never remove them in the first place [79]. Compounded by immense barriers to obtaining documentation, food, stable housing, and other necessities [80–89], legislative changes alone are likely to be insufficient in bolstering voter turnout amongst individuals with felony convictions – mitigating administrative burden to meaningfully improve turnout and strengthen democracy is vital [87,89].

Collectively, this evidence makes salient the need to devote effort to future research and better our understanding of the extent to which the Democratic Party’s messaging may be decreasing solidarity by constructing which groups are “deserving” or not, and what the long-term implications of this are for their ability to effectively represent the needs of their constituents. When reflecting on the decision to employ this stigmatizing rhetoric in an effort to secure the 2024 Presidential Election, the Democratic Party made its choice, perhaps at the expense of 77 million individuals with criminal records [90] throughout the country: was it worth it?

## Supporting information

S1 File Appendix A: Advertisement Support – Individual Item Analysis, Appendix B: Predictive Factors of Outcome Variables. (DOCX)
