# Supplementary material for: Lose the label: Messaging effects on Democrats’ perceptions of individuals with criminal records and representation
Source: PLoS One. 2026 Aug 27;21(8):e0355933. doi: 10.1371/journal.pone.0355933 (PMC13521416; doi:10.1371/journal.pone.0355933)
Supplement: Supplementary file 1 [file pone.0355933.s001.docx]

**Appendix A: Advertisement Support – Individual Item Analysis**

|  | **Effective Advertisement** | | **Support the Use** | |
| --- | --- | --- | --- | --- |
|  | *T* only | *T* + ***X*** | *T* only | *T* + ***X*** |
| Coefficients | -0.055 | -0.048 | -0.279 | -0.271 |
| Robust S.E. | 0.049 | 0.049 | 0.049 | 0.049 |
| *p*-value | 0.262 | 0.328 | < 0.001 | < 0.001 |
| *N* | 2,439 | | 2,439 | |

**Appendix B: Predictive Factors of Outcome Variables**

| **Variable** | **Ad Support** | **Perception** | **Complexity** | **Motives** | **Quality** | **Value** |
| --- | --- | --- | --- | --- | --- | --- |
| (Intercept) | -0.139 (0.160) | 0.363* (0.184) | 0.060 (0.150) | -1.076*** (0.149) | -0.979*** (0.179) | -0.239 (0.190) |
| System Impacted | -0.016 (0.051) | 0.296*** (0.052) | 0.029 (0.053) | -0.030 (0.048) | -0.042 (0.051) | -0.015 (0.053) |
| Man | 0.059 (0.140) | -0.153 (0.172) | -0.141 (0.135) | 0.472*** (0.140) | 0.555*** (0.168) | -0.018 (0.186) |
| Woman | 0.154 (0.138) | -0.278 (0.171) | -0.097 (0.133) | 0.511*** (0.138) | 0.630*** (0.167) | 0.038 (0.185) |
| Non-binary | 0.051 (0.188) | 0.150 (0.225) | -0.085 (0.217) | 0.020 (0.192) | 0.036 (0.227) | -0.008 (0.243) |
| American Indian or Alaskan Native | 0.234 (0.178) | 0.013 (0.168) | 0.017 (0.154) | -0.138 (0.148) | -0.101 (0.163) | 0.257 (0.140) |
| Asian or Asian American | 0.082 (0.073) | -0.183** (0.068) | 0.017 (0.073) | -0.162* (0.070) | -0.173* (0.071) | -0.019 (0.070) |
| Black or African American | -0.022 (0.059) | -0.199*** (0.057) | -0.070 (0.060) | 0.177** (0.059) | 0.139* (0.057) | -0.064 (0.059) |
| Hispanic or Latinx | 0.148* (0.072) | -0.158* (0.079) | -0.020 (0.079) | -0.037 (0.076) | -0.002 (0.075) | -0.067 (0.080) |
| Native Hawaiian, Pacific Islander, or Prefer not to State | 0.306 (0.162) | -0.100 (0.190) | 0.020 (0.173) | 0.599** (0.184) | 0.267 (0.160) | 0.181 (0.177) |
| Age | 0.002 (0.001) | -0.001 (0.001) | -0.000 (0.001) | 0.014*** (0.001) | 0.011*** (0.001) | 0.004* (0.001) |
| Racial attitudes index | 0.191*** (0.023) | 0.125*** (0.021) | 0.094*** (0.021) | 0.012 (0.022) | 0.079*** (0.023) | 0.153*** (0.023) |
| Some college | 0.039 (0.077) | -0.147 (0.082) | 0.106 (0.080) | -0.088 (0.078) | -0.080 (0.076) | 0.088 (0.078) |
| 2-year degree | -0.017 (0.092) | -0.143 (0.094) | 0.065 (0.093) | 0.018 (0.089) | -0.073 (0.090) | 0.135 (0.092) |
| 4-year degree | -0.109 (0.072) | -0.135 (0.074) | 0.068 (0.073) | -0.016 (0.072) | -0.095 (0.070) | 0.071 (0.071) |
| Master’s degree or higher | -0.202** (0.078) | -0.105 (0.080) | 0.070 (0.078) | 0.004 (0.078) | -0.104 (0.076) | 0.107 (0.077) |
| Adjusted R^2^ | 0.05 | 0.04 | 0.01 | 0.06 | 0.05 | 0.02 |
|  | * p<0.05, ** p<0.01, *** p<0.001; Robust SE in parentheses | | | | | |
