# Supplementary material for: Lose the label: Messaging effects on Democrats’ perceptions of individuals with criminal records and representation
Source: PLoS One. 2026 Aug 27;21(8):e0355933. doi: 10.1371/journal.pone.0355933 (PMC13521416; doi:10.1371/journal.pone.0355933)
Supplement: Supplementary file 2 [file pone.0355933.s003.docx]

Dr. Erisen & Reviewers,

Thank you very much for the opportunity to revise, “Lose the label: Messaging effects on Democrats’ perceptions of individuals with criminal records & representation.” I am grateful to you and the reviewers for the thoughtful and helpful comments which have been incorporated into these revisions. I agree whole-heartedly that the feedback and comments received are crucial to ensuring that this paper can effectively share results clearly and such that they are not overstated. As requested, line-by-line responses to the reviewer comments are included below.

Thank you again for your time, the opportunity to resubmit this paper!

***Reviewer #1***

**R1-1: I’ll admit to being skeptical of survey experiments carried out on Prolific and other online platforms in recent months. In the past, my concern was over the degree to which survey takers on these platforms had become professionalized, and were overly responsive to experimental cues. Today, my concern is much more about the extent to which bots, powered by new LLM technologies, have infested these platforms; as early as late 2024, surveys of Prolific users showed that more than a third reported using LLM bots to answer questions. Today, I would expect that figure to be much higher. It is also unclear the extent to which Prolific (or other platforms) are capable of taking action against these bots. Very recently – well after the data in this project was collected – Prolific announced that they have tools which they claim are capable of detecting bots in their surveys, but such tools were, of course, not available to the authors.**

**In the text, the authors note that they removed some responses because of these kinds of concerns: I would want to hear a lot more about that, and how they’re addressing current concerns about LLM-powered bots answering surveys on Prolific and similar platforms.**

This concern is very much heard and shared, so thank you for bringing this up. I have expanded upon the procedure that was utilized to identify and remove responses that were not likely to be human, which was purely based on Qualtrics' reCATPCHA score (i.e., responses that have a reCATPCHA score below 0.5 are cause for concern). The limitations include a section that now provides more in-depth review of these concerns - including the 0.50 threshold utilized - as well as the emerging literature on threats that AI agents pose to survey-based research (e.g., Westwood and others). Simultaneously, there is also emerging literature that documents the range of AI response prevalence (and benchmarks using Westwood) to document that Prolific has considerably lower prevalence of AI (6%) relative to other platforms, such as MTurk (41%) (Chen et al., 2026's working paper, "Estimating the threat of AI-agent responding across online survey platforms"). Additionally, much of the literature that documents the potential threats and/or prevalence of generative AI in online surveys focuses specifically on open-text threats, with some exceptions like Westwood. Still, the propensity for the standard online survey taker to utilize AI agents to complete close-ended surveys is not well-understood. So, while there is full agreement about the caution that should be used when interpreting survey-based research, Prolific is likely the most currently reliable platform to utilize, and thus, the magnitude of threats to the validity of results can be reasonably bounded.

**R1-2: In addition, the fact that the experimental manipulation here was so obvious – at least to the kinds of experienced survey takers found on Prolific – makes me concerned about expectancy effects. Since the respondents in two of the three conditions knew, or probably knew, what the study was about, to what extent are the authors concerned that they were simply telling the authors what they believed the authors wanted to hear? How, if at all, can the authors differentiate between such effects and actual effects of the experimental manipulation? I understand that it may be difficult or impossible for the authors to disentangle such effects, but they need to discuss the issue, and how they are dealing with it, in the manuscript.**

Thank you for this point! Manipulation checks were included, I had just failed to add in the summary of results prior. My apologies! This information has been added to the paper and repasted here for ease of reference (both the sections added to the measurement section and in the results). The manipulation check was asked after the narrative condition (so this was included for all arms, but just varied when it was asked/in what order relative to other stimuli.) "Lastly, a manipulation check was included after exposure to the narrative, specifically asking participants to state the extent to which they agreed with the statement, “I had an emotional reaction to this narrative.” ... "First, analysis of statistical differences on the manipulation check demonstrates the expected effect of the counter-narrative. Specifically, those assigned to the counter-narrative first condition reported significantly higher, on average, agreement to having an emotional response, relative to both the Control group (ATE = -0.156, robust SE = 0.047) and the Trump Ad Alone (ATE = -0.135, robust SE = 0.048), while these latter two groups were not significantly different from one another (difference of 0.021 and robust SE of 0.05). Thus, priming participants with the counter-narrative had the intended effect. This is also useful in providing some evidence against the potential that any subsequent results are largely driven by social desirability bias: if that were true, the emotional reactions to the narrative in the control group would have likely been driven by exposure to the outcome questions that they had answered, which does not appear to have been the case.”

***Reviewer #2***

**R2-1: This manuscript addresses an important and timely question: whether the Democratic Party's use of stigmatizing labels like "convicted criminal" to attack Donald Trump had unintended spillover effects on public perceptions of the 79 million Americans with criminal records. The research is particularly relevant given Democrats' general support for criminal justice reform, raising the possibility of a tension between electoral strategy and core policy commitments. The authors employ an experimental design to test both the negative spillover effects and whether a counter narrative featuring a formerly incarcerated state representative can mitigate these effects. The question is innovative and policy-relevant, and the attempt to identify messaging alternatives is commendable. However, I have substantial concerns about the theoretical framework, research design, measurement validity, and the interpretation of findings that significantly limit the contributions of this work.**

Thank you for the kind words, and completely understood re: concerns. Please see responses below to each specific concern.

**R2-2:The manuscript lacks a clear theoretical model explaining the psychological mechanism through which spillover effects should occur. The authors invoke social categorization theory but do not specify whether the proposed mechanism involves: (a) category priming (making the category "people with criminal records" more salient); (b) affective transfer (negative emotions toward Trump generalizing to the broader category); or (c) cognitive availability (the label becoming more accessible). This ambiguity makes it difficult to evaluate whether the design adequately tests the theoretical proposition.**

**Moreover, there is an internal theoretical contradiction that the manuscript does not address. If Democrats already hold relatively progressive views on criminal justice and less punitive attitudes toward individuals with records (as the authors claim), why would labeling Trump as a "convicted criminal" worsen their perceptions of this population?**

**The manuscript needs to articulate a theory of why this shift would occur. Possible mechanisms might include motivated reasoning (temporarily adopting negative attitudes toward "criminals" to justify attacking Trump), dual-process models (emotional System 1 responses overriding ideological System 2 commitments), or social desirability shifts (feeling licensed to express otherwise-suppressed negative views).**

**The manuscript does not engage with these possibilities. The findings present a paradox that the theoretical framework does not resolve: exposure to the Trump advertisement simultaneously worsened perceptions of individuals with criminal records and improved feelings of representation and beliefs about elected officials' public service motivations. The authors suggest this reflects Prospect Theory with Trump serving as a negative reference point, but this post-hoc interpretation is not developed into a coherent theoretical model.**

**How can Democrats feel better represented (because their officials are not like Trump) while simultaneously adopting more negative views toward a population their party ostensibly supports? The manuscript needs an integrative theoretical framework that explains how these two processes coexist.**

Thank you for each of these points (apologies for lumping a lot of these points together, though they were largely incorporated in tandem.)

First, the review of literature now dives more deeply into the process behind social categorization theory to highlight mechanisms more concretely (i.e., to address the role of saliency and exemplars, and how labels from an individual spillover to a group). See the section beginning with “Specifically, social categorization theory…” for the several additional paragraphs that incorporate this and the other changes from this comment in full.

Specific to the contradiction between progressive policy attitudes not aligning with perceptions of the individuals who benefit from these policies, this section now includes key references to separate (though still connected) domains from the criminal justice system, namely the social safety net. The disconnect between perceptions of beneficiaries of the social safety net and support for policies themselves is pronounced and connected with racial attitudes, ideology, and other factors (not unlike the case of perceptions of individuals with records.) Thus, this section also ties in motivated reasoning as a mechanism through which individuals may hold negative views of individuals while supporting policies that benefit them.

**R2-3: Additionally, the theoretical framework does not address temporal dynamics or persistence. Social categorization theory typically concerns relatively stable cognitive structures, yet the study involves a single, brief exposure to a stimulus. Is this hypothesized to change actual categorization schemas, or is it merely a temporary priming effect? The distinction matters substantially for interpreting the policy implications.**

Thank you for this point! I very much agree about the importance of highlighting this issue concretely in relation to policy recommendations. Thus, the implications section dives into the perspective-getting work and its durability more deeply (specific to the counter-narrative) and the need to study this in future work, as it is indeed essential for developing effective policy.

**R2-4: Research design: The most significant design limitation, which the authors acknowledge but underestimate, is the absence of a full factorial design. The study lacks a "Counter Narrative Only" condition, making it impossible to isolate the effect of the counter narrative from its interaction with the advertisement. All comparisons between the Counter Narrative First group and the Trump Ad Alone group are confounded: we cannot determine whether observed differences reflect (a) the counter narrative neutralizing the ad's effects, (b) the counter narrative alone changing perceptions with the ad having no additional effect, or (c) a unique interactive effect of experiencing both stimuli in sequence. This is not a minor limitation. It fundamentally constrains causal inference about the counter-narrative's effectiveness.**

Thank you very much for this point of feedback! I completely agree and acknowledge this limitation. The omission of the counter-narrative-only arm was driven by binding budget constraints, and I have largely revised and softened the interpretation of results throughout the paper, as will be especially apparent in the conclusion. I agree this is not a minor limitation.

The purpose of this work was to encourage other academics (ideally those with more readily available and robust research funds than myself!) to take up the questions raised here and investigate them with deserved rigor. I hope the paper is read in that spirit: not definitive or standalone evidence, but as an invitation for more empirical attention. The limitations you have identified are precisely the kinds of gaps I hope future work will fill, and I would be delighted to see others build on, challenge, or of course, even overturn these preliminary findings. I have tried to frame the contribution accordingly in the revised manuscript (including in the abstract itself), and I am grateful for feedback that has helped sharpen that framing.

**R2-5: The survey design is highly transparent: participants see an advertisement from their own party attacking Trump, immediately followed by questions about perceptions of people with criminal records. The connection is obvious. This problem is particularly acute in the counter narrative first condition, where participants: (1) read about a representative complaining about the "criminal" label; (2) see the Trump advertisement using exactly that label; and (3) answer questions about perceptions of people with criminal records. The sequence essentially signals the "correct" response. The authors should have included attention checks and comprehension questions about the stimuli, as well as measures of perceived demand.**

I very much understand and acknowledge the concern here, and I appreciate the critique! Attention checks were included at the beginning of the survey, though not in the middle (largely, again, as a result of budget constraints. Including an attention check in the middle or end would require siphoning the sample further rather than rejecting submissions outright or requesting returns, per Prolific’s user agreement, given the survey length.) The analysis of manipulation checks, I believe, helps (but does not completely ameliorate) these concerns. Results of these manipulation checks are described in terms of measurement in the methods section and summarized in regard to the results in the first paragraph of the “main results” section (and more conveniently, in the response to R2-12).

**R2-6: The survey was fielded on July 12-13, 2025—eight months after the November 2024 election, and after Trump had already won. This completely changes the psychological context. Participants are not evaluating the advertisement as a real-time campaign message but retrospectively, with full knowledge of the election outcome. This likely introduces hindsight bias, post-election frustration or anger, and a different emotional landscape than would exist during an active campaign. The findings simply cannot be generalized to the relevant real world context of campaign messaging effects.**

Agreed! This has been acknowledged in the limitations and directions for future research section. The first half of the conclusion, in fact, has been largely rewritten as a result of this (and related) comments.

**R2-7: The Trump advertisement stimulus confounds visual and textual elements: participants see Trump's mugshot with the text "CONVICTED CRIMINAL" superimposed. The authors acknowledge they cannot separate these effects, but this is a substantial limitation. Mugshots themselves are stigmatizing, Trump's mugshot was widely recognized and highly iconic, and the visual element may carry independent effects. Without a condition that presents only the text or only the image, we cannot identify what drives the observed effects.**

Very much understood! This was further and more prominently emphasized as a limitation (see “Additionally, as the advertisement…”), and this is precisely why the framing of the effect is the advertisement. I have thus also adjusted language to soften interpretation and framing of findings to more broadly encompass stigmatization outside of simply the label to also include the possibility that the image drives these results (i.e., “The purpose of this paper was to identify the potential for spillover harm that may have been caused in propagating stigmatizing labels ***(and/or images)*** on Trump, encouraging further inquiry.”)

**R2-8: Participants experienced a single exposure to the advertisement, whereas real campaign targets would experience repeated exposure across multiple platforms. Cumulative effects may differ substantially from single-exposure effects—either amplifying through repetition or diminishing through habituation.**

Thank you for highlighting this! The conclusion now explicitly calls this out as a limitation and an intended item for future research (see the paragraph beginning with “Specifically, the single time-point…”).

**Another issues, the counter-narrative's content may actually reinforce rather than challenge deservingness narratives. The text states: "Jackson said there is a clear difference between a formerly incarcerated person who sought rehabilitation, served their time and was released, and Trump." This creates a binary between "good criminals" (like Jackson, who are deserving of redemption) and "bad criminals" (like Trump, who are not). Rather than challenging stigma toward people with criminal records broadly, this narrative simply adds a new category of deservingness. The effect may not be reducing stigma but creating a new subcategory of acceptable system-involved individuals.**

This is an important point - evidence would agree that there are, in fact, different typologies of individuals with criminal records, with much of the variation in perceptions individuals with criminal records driven by ideology, personal exposure, and the extent to which individuals see those with criminal records as being capable of redemption (see citations, a few have been added, e.g., Maruna’s work). This literature has been incorporated into both the justification of the counter-narrative and discussion of the results. While currently, it is out of the scope of this paper to definitively answer this, it has been identified as an opportunity for future research.

**R2-9: Measurement: Social desirability concerns are severe. The items are highly transparent:**

**- "People with criminal records deserve to have the same level of opportunity for civic participation..."**

**- "Most people with criminal records do not positively contribute to their communities..."**

**Participants can easily identify the "correct" answer, particularly after reading the counter-narrative that explicitly discusses stigma and representation. The authors should have included implicit measures or less transparent items.**

Thank you for this comment! It is certainly heard and acknowledged that social desirability bias is at play. Still, the questions posed (and the resulting distribution of responses) do demonstrate that a non-trivial proportion of the sample of Democrats provided responses that were somewhat impervious to concerns about social desirability and that align with robust, larger-scale surveys on such topics, such as the General Social Survey. In this sample, 12% did not agree that there is a great deal discrimination against people of color, and 17% did not agree that people of color have gotten less than they deserve. Though certainly not a precise comparison, GSS asks questions specific to Black Americans, (e.g., "overcoming prejudice without favors"), to which 29% of Democrats did not agree with this statement, so this is not considerably out of lin. Specific to the perceptions questions, these are not far out of line with other studies on perceptions of individuals with a specific focus on measuring stigma. For example, in studies of individuals who receive public benefits, questions often utilize a frame of "Most people who receive [a particular program of interest]" and ask participants to indicate their perceptions on the extent to which recipients "are poor because of the choices they make," "waste money when they have it", and "do not contribute their fair share to society" (Lasky-Fink et al., 2026, not cited in manuscript, but linked [here](https://www.hks.harvard.edu/publications/stigma-and-social-safety-net)).

**R2-10: The advertisement support index comprises only two items: (1) "This is an effective advertisement" and (2) "I support use of this advertisement." These tap fundamentally different constructs, instrumental effectiveness versus normative approval. A participant could believe the advertisement is effective (i.e., will help win the election) while opposing its use on moral grounds. Collapsing these into a single index obscures this important distinction.**

Thank you for this really important note and critique! I have added supplemental analyses that show the effect of the counter-narrative with advertisement arm, relative to the advertisement only group. The summarized results are added to the Results section, with the additional table included within appendices. Both coefficients are negative, demonstrating that the counter-narrative decreased individuals' support and perceived effectiveness of the advertisement: "I support use of this advertisement" and "This is an effective advertisement", respectively. While both coefficients are negative, the results are driven primarily by the support item (-0.27, relative to -0.05). Within the paper, this content has been added within the paragraph beginning with "Starting with H1A"

**R2-11: Analysis: The heterogeneity analysis by system-impact status (Figure 4) examines a relatively small subsample (about 20% of the sample), limiting statistical power. Moreover, this analysis is supplemental and not pre-registered? increasing the risk of false positives. The authors report heterogeneity for only some outcomes, was heterogeneity tested for all measures and reported selectively? This should be clarified.**

Thank you for this point! The heterogeneity by system-impact status was pre-registered, but framed as exploratory for the precise reason you identified (i.e., limited statistical power). Heterogeneity was only tested for the perceptions measure (index and individual items) to limit the possibility of false positives. If subsequent revisions are requested, and heterogeneity on indices is desired, this can be added (with the same caveat of analyses being exploratory in nature).

**R2-12: The authors did not include clear manipulation checks? How do we know whether participants: (a) actually read the counter narrative (b) understood its content or (c) remembered it when answering outcome questions. Given that the text is relatively long and the survey was administered online (where participants may rush through), this is a serious omission. Without manipulation checks, we cannot be certain the treatment was received as intended.**

Thank you for this point! Manipulation checks were included, I had just failed to add in the summary of results prior. My apologies! This information has been added to the paper and repasted here for ease of reference (both the sections added to the measurement section and in the results). The manipulation check was asked after the narrative condition (so this was included for all arms, but just varied when it was asked/in what order relative to other stimuli.) "Lastly, a manipulation check was included after exposure to the narrative, specifically asking participants to state the extent to which they agreed with the statement, “I had an emotional reaction to this narrative.” ... "First, analysis of statistical differences on the manipulation check demonstrates the expected effect of the counter-narrative. Specifically, those assigned to the counter-narrative first condition reported significantly higher, on average, agreement to having an emotional response, relative to both the Control group (ATE = -0.156, robust SE = 0.047) and the Trump Ad Alone (ATE = -0.135, robust SE = 0.048), while these latter two groups were not significantly different from one another (difference of 0.021 and robust SE of 0.05). Thus, priming participants with the counter-narrative had the intended effect. This is also useful in providing some evidence against the potential that any subsequent results are largely driven by social desirability bias: if that were true, the emotional reactions to the narrative in the control group would have likely been driven by exposure to the outcome questions that they had answered, which does not appear to have been the case.”

**R2-13: To sum up, here are my suggestion for the authors:**

1. **Develop a clear, explicit theoretical model of the psychological mechanism underlying spillover effects, addressing why Democrats would adopt more negative attitudes toward a population their party supports.**
2. **Conduct a replication study with a full factorial design that includes a Counter Narrative Only condition, allowing clean identification of the counter narrative's effect.**
3. **Field the survey during an actual campaign (or simulate realistic campaign conditions) rather than eight months after the election, when the psychological context is entirely different.**
4. **Include manipulation checks to verify that participants engaged with and understood the stimuli.**
5. **Add less transparent, more implicit measures of attitudes toward people with criminal records to reduce social desirability bias.**
6. **Apply appropriate corrections for multiple comparisons.**
7. **Revise the normative claims to align with what the evidence actually demonstrates, acknowledging that short term survey responses do not directly translate to real world harm.**
8. **Engage more deeply with the strategic and ethical tradeoffs involved in campaign messaging decisions.**

1. Thank you! See comment R2-4.

2. As a result of resource constraints, I have not undergone this task; however, I have considerably edited and softened the discussion to emphasize the importance and need for this work.

3. Related to point 2, I have identified this for future work (as these resource constraints are still binding, and this paper is intended to encourage future work.)

4. The results of the manipulation checks are in the paper now - thank you!

5. See comment R2-9.

6. Thank you! This was buried a bit in the paper, so I have also added it to table notes for both (the public-serving motives outcome is the only one that would not be significant after multiple comparisons, of outcomes with significant differences.)

7. This has been revised heavily in the limitations section!

8. The implications section now spends more time talking about the importance of acknowledging the charges, though specifically notes that this paper seeks to encourage researchers to explore this space further to understand the extent to which these trade-offs are considerable or if there may be strategic benefits to ethical approaches in navigating these issues.
