# Supplementary material for: Lose the label: Messaging effects on Democrats’ perceptions of individuals with criminal records and representation
Source: PLoS One. 2026 Aug 27;21(8):e0355933. doi: 10.1371/journal.pone.0355933 (PMC13521416; doi:10.1371/journal.pone.0355933)
Supplement: Supplementary file 3 [file pone.0355933.s004.docx]

Dr. Erisen & Reviewers,

Thank you very much for the opportunity to provide further revisions of, “Lose the label: Messaging effects on Democrats’ perceptions of individuals with criminal records & representation.” I am grateful to you and the reviewers for the thoughtful and helpful comments which have been incorporated into these revisions.

Thank you again for your time and the opportunity to resubmit this paper again!

***Reviewer #1***

**R1-1: I believe that the authors have adequately responded to my comments from the first round of reviews (though it seems like the other reviewer had more serious issues).**

Thank you!

***Reviewer #2***

N/A

***Reviewer #3***

**R3-1: This is a new review of a previously revised manuscript. I read the author's responses to the original reviews and will leave it to the reviewers and editors to determine if the manuscript was appropriately revised. As the manuscript currently stands, I have two related critiques.**

**First, there is little discussion of the ample literature on negative campaign advertising. I realize that this manuscript is not directly interested in negative campaigning and is more interested in the effects of language (and counter-narratives). There is, however, much that can be taken from this literature, starting with work from Ansolabehere and Iyengar in the mid-1990s, on how ads like the ones emphasizing Trump's interactions with the justice system, affect people's processing of related information and their behavior. At the very least, this literature can help better answer some of the speculative questions in the discussion and concluding sections, and better inform the presentation of theory in the front end of the manuscript. I include this critique not simply to say that there are other papers that should be cited here. I include it because this experiment was conducted in the context of, and proports to help us understand, reactions to an actual negative campaign ad. The context matters. Respondents were not presented with a conversation, or a speech, or some other type of interaction; they were presented with a campaign ad used by the Democratic Party in the 2024 election. That has the potential to lead to other cognitive effects beyond the message itself that are relevant to the theoretical arguments, including what effective counter-narratives look like and how they would work in real world conditions.**

Thank you for this excellent point! The introduction and conclusion sections have now both been updated. The introduction includes a brief discussion of the negative campaign literature, while the discussion notes the importance of considering the psychological effects of negative campaign ads in the interpretation of results (and as a limitation, given the constraints on not being able to run the full factorial.) Each section’s additions have been included below:

*Introduction*

“The more negative angle for campaign advertisements and rhetoric is important to note: prior evidence on the effects of negative campaign advertisements suggests that such advertisements tend to be utilized less by those leading in the polls [4-5] and that Democrats tend to respond more to positive ads, compared to Republicans.[6] Additionally, meta-analytic evidence does not empirically support the use of negative campaign advertisements to aid election success.[7]”

*Conclusion*

This is important, as utilizing a negative campaign advertisement could moderate the effect of the counter-narrative perspective intervention, depending upon one’s personality traits, amongst other potential factors.[66]

**R3-2: Second, and perhaps more specific to an aspect of the literature, there is no attention to sophistication or related concepts. From Table 1, I assume that the survey did not include items that could be used to gauge, even rough levels of sophistication. Education is split between less than a 4-year degree and at least a 4-year degree. Education is, however, only a correlate of sophistication. I recognize that, as an experiment, the core analysis simply compares the treatment to the control (or here, the double-treatment to the single -treatment). Given the context, that's a missed opportunity, especially with outcome questions relating to political efficacy. I also recognize that, assuming these items were not included, nothing can be done empirically about this now, but it would be worth the author using the literature to speculate about what the potential effects of sophistication are here.**

Thank you also for making this point! Education was measured as a categorical variable with 7 categories: a) less than high school; b) high school; c) some college; d) 2-year degree; e) 4-year degree; f) master’s or higher; and g) prefer not state. Note that only one individual noted prefer not to state, and less than 10 indicated having less than a high school degree, so these values were collapsed into a single category of having a high school degree or less, which serves as the reference category. The full results table has been included Appendix B to complement the summarized Table 4 included in the main text. Most education levels were not a significant predictor of any outcome measure (using high school education or less as the reference), with exception of the advertisement support, where those with a master’s degree or higher reported significantly less support for the advertisement, on average. Table 4 has been updated with the education categories instead of the binarized version of education level.

**R3-3: I also want to add that I am not an expert in modern experimental research. More specifically, I am not up on the use of platforms like Prolific. That said, the empirical analyses seem perfectly reasonable to me and the conclusions accurately reflect the results as presented.**

Thank you for taking the time to review! Your effort and feedback are appreciated!
