# Supplementary material for: Lose the label: Messaging effects on Democrats’ perceptions of individuals with criminal records and representation
Source: PLoS One. 2026 Aug 27;21(8):e0355933. doi: 10.1371/journal.pone.0355933 (PMC13521416; doi:10.1371/journal.pone.0355933)
Supplement: Peer review history

# Peer review: Decision Letter 0

**DOI:** 10.1371/journal.pone.0355933.r001

**Authors:** Cengiz Erisen

11 Mar 2026

PLOS One

Dear Dr. Harney,

Thank you for submitting your manuscript to PLOS ONE. After careful consideration, we feel that it has merit but does not fully meet PLOS ONE’s publication criteria as it currently stands. Therefore, we invite you to submit a revised version of the manuscript that addresses the points raised during the review process.

Both reviewers raise substantial concerns regarding the study’s theoretical clarity, research design, and data reliability. In particular, first, the manuscript lacks a clearly articulated theoretical mechanism explaining how stigmatizing campaign messages would produce spillover effects toward individuals with criminal records. To address this gap, I strongly recommend checking out the literature about motivated reasoning and incongruent information behavior (e.g., D. Redlawsk’s or M. Lodge’s work). Also, earlier work in political science on anger and defensive attitudes would be quite useful. Second, important methodological limitations such as the absence of a full factorial design, transparent experimental setup, and the potential influence of demand characteristics make it difficult to draw clear causal conclusions. These methodological concerns require attention and clarifications. Finally, as R1 raises, the reliability of the Prolific sample requires further explanation.

Please submit your revised manuscript by Apr 24 2026 11:59PM. If you will need more time than this to complete your revisions, please reply to this message or contact the journal office at plosone@plos.org. When you're ready to submit your revision, log on to https://www.editorialmanager.com/pone/ and select the 'Submissions Needing Revision' folder to locate your manuscript file.

Please include the following items when submitting your revised manuscript:

- A letter that responds to each point raised by the academic editor and reviewer(s). You should upload this letter as a separate file labeled 'Response to Reviewers'.
- A marked-up copy of your manuscript that highlights changes made to the original version. You should upload this as a separate file labeled 'Revised Manuscript with Track Changes'.
- An unmarked version of your revised paper without tracked changes. You should upload this as a separate file labeled 'Manuscript'.

If you would like to make changes to your financial disclosure, please include your updated statement in your cover letter. Guidelines for resubmitting your figure files are available below the reviewer comments at the end of this letter.

If applicable, we recommend that you deposit your laboratory protocols in protocols.io to enhance the reproducibility of your results. Protocols.io assigns your protocol its own identifier (DOI) so that it can be cited independently in the future. For instructions see: https://journals.plos.org/plosone/s/submission-guidelines#loc-laboratory-protocols. Additionally, PLOS ONE offers an option for publishing peer-reviewed Lab Protocol articles, which describe protocols hosted on protocols.io. Read more information on sharing protocols at https://plos.org/protocols?utm_medium=editorial-email&utm_source=authorletters&utm_campaign=protocols.

We look forward to receiving your revised manuscript.

Kind regards,

Cengiz Erisen

Academic Editor

PLOS One

Journal Requirements:

When submitting your revision, we need you to address these additional requirements.

1.Please ensure that your manuscript meets PLOS ONE's style requirements, including those for file naming. The PLOS ONE style templates can be found at

https://journals.plos.org/plosone/s/file?id=wjVg/PLOSOne_formatting_sample_main_body.pdf and

https://journals.plos.org/plosone/s/file?id=ba62/PLOSOne_formatting_sample_title_authors_affiliations.pdf

2. Please include your tables as part of your main manuscript and remove the individual files. Please note that supplementary tables (should remain/ be uploaded) as separate "supporting information" files.

3. Please note that your Data Availability Statement is currently missing the repository. If your manuscript is accepted for publication, you will be asked to provide these details on a very short timeline. We therefore suggest that you provide this information now, though we will not hold up the peer review process if you are unable.

4. Please include your full ethics statement in the ‘Methods’ section of your manuscript file. In your statement, please include the full name of the IRB or ethics committee who approved or waived your study, as well as whether or not you obtained informed written or verbal consent. If consent was waived for your study, please include this information in your statement as well.

5. We note that Figure 2 in your submission contain copyrighted images. All PLOS content is published under the Creative Commons Attribution License (CC BY 4.0), which means that the manuscript, images, and Supporting Information files will be freely available online, and any third party is permitted to access, download, copy, distribute, and use these materials in any way, even commercially, with proper attribution. For more information, see our copyright guidelines: http://journals.plos.org/plosone/s/licenses-and-copyright.

We require you to either (1) present written permission from the copyright holder to publish these figures specifically under the CC BY 4.0 license, or (2) remove the figures from your submission:

1. You may seek permission from the original copyright holder of Figure 2 to publish the content specifically under the CC BY 4.0 license.

We recommend that you contact the original copyright holder with the Content Permission Form (http://journals.plos.org/plosone/s/file?id=7c09/content-permission-form.pdf) and the following text:

“I request permission for the open-access journal PLOS ONE to publish XXX under the Creative Commons Attribution License (CCAL) CC BY 4.0 (http://creativecommons.org/licenses/by/4.0/). Please be aware that this license allows unrestricted use and distribution, even commercially, by third parties. Please reply and provide explicit written permission to publish XXX under a CC BY license and complete the attached form.”

Please upload the completed Content Permission Form or other proof of granted permissions as an "Other" file with your submission.

In the figure caption of the copyrighted figure, please include the following text: “Reprinted from [ref] under a CC BY license, with permission from [name of publisher], original copyright [original copyright year].”

2. If you are unable to obtain permission from the original copyright holder to publish these figures under the CC BY 4.0 license or if the copyright holder’s requirements are incompatible with the CC BY 4.0 license, please either i) remove the figure or ii) supply a replacement figure that complies with the CC BY 4.0 license. Please check copyright information on all replacement figures and update the figure caption with source information. If applicable, please specify in the figure caption text when a figure is similar but not identical to the original image and is therefore for illustrative purposes only.

6. If the reviewer comments include a recommendation to cite specific previously published works, please review and evaluate these publications to determine whether they are relevant and should be cited. There is no requirement to cite these works unless the editor has indicated otherwise.

[Note: HTML markup is below. Please do not edit.]

Reviewers' comments:

Reviewer's Responses to Questions

**Comments to the Author**

1. Is the manuscript technically sound, and do the data support the conclusions?

Reviewer #1: Yes

Reviewer #2: Yes

**********

2. Has the statistical analysis been performed appropriately and rigorously?

Reviewer #1: Yes

Reviewer #2: Yes

**********

3. Have the authors made all data underlying the findings in their manuscript fully available?

The PLOS Data policy

Reviewer #1: Yes

Reviewer #2: Yes

**********

4. Is the manuscript presented in an intelligible fashion and written in standard English?

Reviewer #1: Yes

Reviewer #2: Yes

**********

Reviewer #1: I’ll admit to being skeptical of survey experiments carried out on Prolific and other online platforms in recent months. In the past, my concern was over the degree to which survey takers on these platforms had become professionalized, and were overly responsive to experimental cues. Today, my concern is much more about the extent to which bots, powered by new LLM technologies, have infested these platforms; as early as late 2024, surveys of Prolific users showed that more than a third reported using LLM bots to answer questions. Today, I would expect that figure to be much higher. It is also unclear the extent to which Prolific (or other platforms) are capable of taking action against these bots. Very recently – well after the data in this project was collected – Prolific announced that they have tools which they claim are capable of detecting bots in their surveys, but such tools were, of course, not available to the authors.

In the text, the authors note that they removed some responses because of these kinds of concerns: I would want to hear a lot more about that, and how they’re addressing current concerns about LLM-powered bots answering surveys on Prolific and similar platforms.

In addition, the fact that the experimental manipulation here was so obvious – at least to the kinds of experienced survey takers found on Prolific – makes me concerned about expectancy effects. Since the respondents in two of the three conditions knew, or probably knew, what the study was about, to what extent are the authors concerned that they were simply telling the authors what they believed the authors wanted to hear? How, if at all, can the authors differentiate between such effects and actual effects of the experimental manipulation? I understand that it may be difficult or impossible for the authors to disentangle such effects, but they need to discuss the issue, and how they are dealing with it, in the manuscript.

My other concerns are outside of the bounds of what PLOS asks for from a reviewer, so I will forego them at this time.

Reviewer #2: This manuscript addresses an important and timely question: whether the Democratic Party's use of stigmatizing labels like "convicted criminal" to attack Donald Trump had unintended spillover effects on public perceptions of the 79 million Americans with criminal records. The research is particularly relevant given Democrats' general support for criminal justice reform, raising the possibility of a tension between electoral strategy and core policy commitments. The authors employ an experimental design to test both the negative spillover effects and whether a counter narrative featuring a formerly incarcerated state representative can mitigate these effects. The question is innovative and policy-relevant, and the attempt to identify messaging alternatives is commendable.

However, I have substantial concerns about the theoretical framework, research design, measurement validity, and the interpretation of findings that significantly limit the contributions of this work.

Theoretical framework: The manuscript lacks a clear theoretical model explaining the psychological mechanism through which spillover effects should occur. The authors invoke social categorization theory but do not specify whether the proposed mechanism involves: (a) category priming (making the category "people with criminal records" more salient); (b) affective transfer (negative emotions toward Trump generalizing to the broader category); or (c) cognitive availability (the label becoming more accessible). This ambiguity makes it difficult to evaluate whether the design adequately tests the theoretical proposition.

Moreover, there is an internal theoretical contradiction that the manuscript does not address. If Democrats already hold relatively progressive views on criminal justice and less punitive attitudes toward individuals with records (as the authors claim), why would labeling Trump as a "convicted criminal" worsen their perceptions of this population? The manuscript needs to articulate a theory of why this shift would occur. Possible mechanisms might include motivated reasoning (temporarily adopting negative attitudes toward "criminals" to justify attacking Trump), dual-process models (emotional System 1 responses overriding ideological System 2 commitments), or social desirability shifts (feeling licensed to express otherwise-suppressed negative views). The manuscript does not engage with these possibilities.

The findings present a paradox that the theoretical framework does not resolve: exposure to the Trump advertisement simultaneously worsened perceptions of individuals with criminal records and improved feelings of representation and beliefs about elected officials' public service motivations. The authors suggest this reflects Prospect Theory with Trump serving as a negative reference point, but this post-hoc interpretation is not developed into a coherent theoretical model. How can Democrats feel better represented (because their officials are not like Trump) while simultaneously adopting more negative views toward a population their party ostensibly supports? The manuscript needs an integrative theoretical framework that explains how these two processes coexist.

Additionally, the theoretical framework does not address temporal dynamics or persistence. Social categorization theory typically concerns relatively stable cognitive structures, yet the study involves a single, brief exposure to a stimulus. Is this hypothesized to change actual categorization schemas, or is it merely a temporary priming effect? The distinction matters substantially for interpreting the policy implications.

Research design: The most significant design limitation, which the authors acknowledge but underestimate, is the absence of a full factorial design. The study lacks a "Counter Narrative Only" condition, making it impossible to isolate the effect of the counter narrative from its interaction with the advertisement. All comparisons between the Counter Narrative First group and the Trump Ad Alone group are confounded: we cannot determine whether observed differences reflect (a) the counter narrative neutralizing the ad's effects, (b) the counter narrative alone changing perceptions with the ad having no additional effect, or (c) a unique interactive effect of experiencing both stimuli in sequence. This is not a minor limitation. It fundamentally constrains causal inference about the counter-narrative's effectiveness.

The survey design is highly transparent: participants see an advertisement from their own party attacking Trump, immediately followed by questions about perceptions of people with criminal records. The connection is obvious. This problem is particularly acute in the counter narrative first condition, where participants: (1) read about a representative complaining about the "criminal" label; (2) see the Trump advertisement using exactly that label; and (3) answer questions about perceptions of people with criminal records. The sequence essentially signals the "correct" response. The authors should have included attention checks and comprehension questions about the stimuli, as well as measures of perceived demand.

The survey was fielded on July 12-13, 2025—eight months after the November 2024 election, and after Trump had already won. This completely changes the psychological context. Participants are not evaluating the advertisement as a real-time campaign message but retrospectively, with full knowledge of the election outcome. This likely introduces hindsight bias, post-election frustration or anger, and a different emotional landscape than would exist during an active campaign. The findings simply cannot be generalized to the relevant real world context of campaign messaging effects.

The Trump advertisement stimulus confounds visual and textual elements: participants see Trump's mugshot with the text "CONVICTED CRIMINAL" superimposed. The authors acknowledge they cannot separate these effects, but this is a substantial limitation. Mugshots themselves are stigmatizing, Trump's mugshot was widely recognized and highly iconic, and the visual element may carry independent effects. Without a condition that presents only the text or only the image, we cannot identify what drives the observed effects.

Participants experienced a single exposure to the advertisement, whereas real campaign targets would experience repeated exposure across multiple platforms. Cumulative effects may differ substantially from single-exposure effects—either amplifying through repetition or diminishing through habituation.

Another issues, the counter-narrative's content may actually reinforce rather than challenge deservingness narratives. The text states: "Jackson said there is a clear difference between a formerly incarcerated person who sought rehabilitation, served their time and was released, and Trump." This creates a binary between "good criminals" (like Jackson, who are deserving of redemption) and "bad criminals" (like Trump, who are not). Rather than challenging stigma toward people with criminal records broadly, this narrative simply adds a new category of deservingness. The effect may not be reducing stigma but creating a new subcategory of acceptable system-involved individuals.

Measurement: Social desirability concerns are severe. The items are highly transparent:

- "People with criminal records deserve to have the same level of opportunity for civic participation..."

- "Most people with criminal records do not positively contribute to their communities..."

Participants can easily identify the "correct" answer, particularly after reading the counter-narrative that explicitly discusses stigma and representation. The authors should have included implicit measures or less transparent items.

The advertisement support index comprises only two items: (1) "This is an effective advertisement" and (2) "I support use of this advertisement." These tap fundamentally different constructs, instrumental effectiveness versus normative approval. A participant could believe the advertisement is effective (i.e., will help win the election) while opposing its use on moral grounds. Collapsing these into a single index obscures this important distinction.

Analysis: The heterogeneity analysis by system-impact status (Figure 4) examines a relatively small subsample (about 20% of the sample), limiting statistical power. Moreover, this analysis is supplemental and not pre-registered? increasing the risk of false positives. The authors report heterogeneity for only some outcomes, was heterogeneity tested for all measures and reported selectively? This should be clarified.

The authors did not include clear manipulation checks? How do we know whether participants: (a) actually read the counter narrative (b) understood its content or (c) remembered it when answering outcome questions. Given that the text is relatively long and the survey was administered online (where participants may rush through), this is a serious omission. Without manipulation checks, we cannot be certain the treatment was received as intended.

To sum up, here are my suggestion for the authors:

1. Develop a clear, explicit theoretical model of the psychological mechanism underlying spillover effects, addressing why Democrats would adopt more negative attitudes toward a population their party supports.

2. Conduct a replication study with a full factorial design that includes a Counter Narrative Only condition, allowing clean identification of the counter narrative's effect.

3. Field the survey during an actual campaign (or simulate realistic campaign conditions) rather than eight months after the election, when the psychological context is entirely different.

4. Include manipulation checks to verify that participants engaged with and understood the stimuli.

5. Add less transparent, more implicit measures of attitudes toward people with criminal records to reduce social desirability bias.

6. Apply appropriate corrections for multiple comparisons.

7. Revise the normative claims to align with what the evidence actually demonstrates, acknowledging that short term survey responses do not directly translate to real world harm.

8. Engage more deeply with the strategic and ethical tradeoffs involved in campaign messaging decisions.

**********

what does this mean?). If published, this will include your full peer review and any attached files.

If you choose “no”, your identity will remain anonymous but your review may still be made public.

**Do you want your identity to be public for this peer review?**  For information about this choice, including consent withdrawal, please see our Privacy Policy

Reviewer #1: **Yes:** Dan Cassino

Reviewer #2: No

**********

[NOTE: If reviewer comments were submitted as an attachment file, they will be attached to this email and accessible via the submission site. Please log into your account, locate the manuscript record, and check for the action link "View Attachments". If this link does not appear, there are no attachment files.]

To ensure your figures meet our technical requirements, please review our figure guidelines: https://journals.plos.org/plosone/s/figures

You may also use PLOS’s free figure tool, NAAS, to help you prepare publication quality figures: https://journals.plos.org/plosone/s/figures#loc-tools-for-figure-preparation.

NAAS will assess whether your figures meet our technical requirements by comparing each figure against our figure specifications.

# Author comment: Author response to Decision Letter 1

**DOI:** 10.1371/journal.pone.0355933.r002

25 Apr 2026

Dr. Erisen & Reviewers,

Thank you very much for the opportunity to revise, “Lose the label: Messaging effects on Democrats’ perceptions of individuals with criminal records & representation.” I am grateful to you and the reviewers for the thoughtful and helpful comments which have been incorporated into these revisions. I agree whole-heartedly that the feedback and comments received are crucial to ensuring that this paper can effectively share results clearly and such that they are not overstated. As requested, line-by-line responses to the reviewer comments are included below.

Thank you again for your time, the opportunity to resubmit this paper!

Reviewer #1

R1-1: I’ll admit to being skeptical of survey experiments carried out on Prolific and other online platforms in recent months. In the past, my concern was over the degree to which survey takers on these platforms had become professionalized, and were overly responsive to experimental cues. Today, my concern is much more about the extent to which bots, powered by new LLM technologies, have infested these platforms; as early as late 2024, surveys of Prolific users showed that more than a third reported using LLM bots to answer questions. Today, I would expect that figure to be much higher. It is also unclear the extent to which Prolific (or other platforms) are capable of taking action against these bots. Very recently – well after the data in this project was collected – Prolific announced that they have tools which they claim are capable of detecting bots in their surveys, but such tools were, of course, not available to the authors.

In the text, the authors note that they removed some responses because of these kinds of concerns: I would want to hear a lot more about that, and how they’re addressing current concerns about LLM-powered bots answering surveys on Prolific and similar platforms.

This concern is very much heard and shared, so thank you for bringing this up. I have expanded upon the procedure that was utilized to identify and remove responses that were not likely to be human, which was purely based on Qualtrics' reCATPCHA score (i.e., responses that have a reCATPCHA score below 0.5 are cause for concern). The limitations include a section that now provides more in-depth review of these concerns - including the 0.50 threshold utilized - as well as the emerging literature on threats that AI agents pose to survey-based research (e.g., Westwood and others). Simultaneously, there is also emerging literature that documents the range of AI response prevalence (and benchmarks using Westwood) to document that Prolific has considerably lower prevalence of AI (6%) relative to other platforms, such as MTurk (41%) (Chen et al., 2026's working paper, "Estimating the threat of AI-agent responding across online survey platforms"). Additionally, much of the literature that documents the potential threats and/or prevalence of generative AI in online surveys focuses specifically on open-text threats, with some exceptions like Westwood. Still, the propensity for the standard online survey taker to utilize AI agents to complete close-ended surveys is not well-understood. So, while there is full agreement about the caution that should be used when interpreting survey-based research, Prolific is likely the most currently reliable platform to utilize, and thus, the magnitude of threats to the validity of results can be reasonably bounded.

R1-2: In addition, the fact that the experimental manipulation here was so obvious – at least to the kinds of experienced survey takers found on Prolific – makes me concerned about expectancy effects. Since the respondents in two of the three conditions knew, or probably knew, what the study was about, to what extent are the authors concerned that they were simply telling the authors what they believed the authors wanted to hear? How, if at all, can the authors differentiate between such effects and actual effects of the experimental manipulation? I understand that it may be difficult or impossible for the authors to disentangle such effects, but they need to discuss the issue, and how they are dealing with it, in the manuscript.

Thank you for this point! Manipulation checks were included, I had just failed to add in the summary of results prior. My apologies! This information has been added to the paper and repasted here for ease of reference (both the sections added to the measurement section and in the results). The manipulation check was asked after the narrative condition (so this was included for all arms, but just varied when it was asked/in what order relative to other stimuli.) "Lastly, a manipulation check was included after exposure to the narrative, specifically asking participants to state the extent to which they agreed with the statement, “I had an emotional reaction to this narrative.” ... "First, analysis of statistical differences on the manipulation check demonstrates the expected effect of the counter-narrative. Specifically, those assigned to the counter-narrative first condition reported significantly higher, on average, agreement to having an emotional response, relative to both the Control group (ATE = -0.156, robust SE = 0.047) and the Trump Ad Alone (ATE = -0.135, robust SE = 0.048), while these latter two groups were not significantly different from one another (difference of 0.021 and robust SE of 0.05). Thus, priming participants with the counter-narrative had the intended effect. This is also useful in providing some evidence against the potential that any subsequent results are largely driven by social desirability bias: if that were true, the emotional reactions to the narrative in the control group would have likely been driven by exposure to the outcome questions that they had answered, which does not appear to have been the case.”

Reviewer #2

R2-1: This manuscript addresses an important and timely question: whether the Democratic Party's use of stigmatizing labels like "convicted criminal" to attack Donald Trump had unintended spillover effects on public perceptions of the 79 million Americans with criminal records. The research is particularly relevant given Democrats' general support for criminal justice reform, raising the possibility of a tension between electoral strategy and core policy commitments. The authors employ an experimental design to test both the negative spillover effects and whether a counter narrative featuring a formerly incarcerated state representative can mitigate these effects. The question is innovative and policy-relevant, and the attempt to identify messaging alternatives is commendable. However, I have substantial concerns about the theoretical framework, research design, measurement validity, and the interpretation of findings that significantly limit the contributions of this work.

Thank you for the kind words, and completely understood re: concerns. Please see responses below to each specific concern.

R2-2:The manuscript lacks a clear theoretical model explaining the psychological mechanism through which spillover effects should occur. The authors invoke social categorization theory but do not specify whether the proposed mechanism involves: (a) category priming (making the category "people with criminal records" more salient); (b) affective transfer (negative emotions toward Trump generalizing to the broader category); or (c) cognitive availability (the label becoming more accessible). This ambiguity makes it difficult to evaluate whether the design adequately tests the theoretical proposition.

Moreover, there is an internal theoretical contradiction that the manuscript does not address. If Democrats already hold relatively progressive views on criminal justice and less punitive attitudes toward individuals with records (as the authors claim), why would labeling Trump as a "convicted criminal" worsen their perceptions of this population?

The manuscript needs to articulate a theory of why this shift would occur. Possible mechanisms might include motivated reasoning (temporarily adopting negative attitudes toward "criminals" to justify attacking Trump), dual-process models (emotional System 1 responses overriding ideological System 2 commitments), or social desirability shifts (feeling licensed to express otherwise-suppressed negative views).

The manuscript does not engage with these possibilities. The findings present a paradox that the theoretical framework does not resolve: exposure to the Trump advertisement simultaneously worsened perceptions of individuals with criminal records and improved feelings of representation and beliefs about elected officials' public service motivations. The authors suggest this reflects Prospect Theory with Trump serving as a negative reference point, but this post-hoc interpretation is not developed into a coherent theoretical model.

How can Democrats feel better represented (because their officials are not like Trump) while simultaneously adopting more negative views toward a population their party ostensibly supports? The manuscript needs an integrative theoretical framework that explains how these two processes coexist.

Thank you for each of these points (apologies for lumping a lot of these points together, though they were largely incorporated in tandem.)

First, the review of literature now dives more deeply into the process behind social categorization theory to highlight mechanisms more concretely (i.e., to address the role of saliency and exemplars, and how labels from an individual spillover to a group). See the section beginning with “Specifically, social categorization theory…” for the several additional paragraphs that incorporate this and the other changes from this comment in full.

Specific to the contradiction between progressive policy attitudes not aligning with perceptions of the individuals who benefit from these policies, this section now includes key references to separate (though still connected) domains from the criminal justice system, namely the social safety net. The disconnect between perceptions of beneficiaries of the social safety net and support for policies themselves is pronounced and connected with racial attitudes, ideology, and other factors (not unlike the case of perceptions of individuals with records.) Thus, this section also ties in motivated reasoning as a mechanism through which individuals may hold negative views of individuals while supporting policies that benefit them.

R2-3: Additionally, the theoretical framework does not address temporal dynamics or persistence. Social categorization theory typically concerns relatively stable cognitive structures, yet the study involves a single, brief exposure to a stimulus. Is this hypothesized to change actual categorization schemas, or is it merely a temporary priming effect? The distinction matters substantially for interpreting the policy implications.

Thank you for this point! I very much agree about the importance of highlighting this issue concretely in relation to policy recommendations. Thus, the implications section dives into the perspective-getting work and its durability more deeply (specific to the counter-narrative) and the need to study this in future work, as it is indeed essential for developing effective policy.

R2-4: Research design: The most significant design limitation, which the authors acknowledge but underestimate, is the absence of a full factorial design. The study lacks a "Counter Narrative Only" condition, making it impossible to isolate the effect of the counter narrative from its interaction with the advertisement. All comparisons between the Counter Narrative First group and the Trump Ad Alone group are confounded: we cannot determine whether observed differences reflect (a) the counter narrative neutralizing the ad's effects, (b) the counter narrative alone changing perceptions with the ad having no additional effect, or (c) a unique interactive effect of experiencing both stimuli in sequence. This is not a minor limitation. It fundamentally constrains causal inference about the counter-narrative's effectiveness.

Thank you very much for this point of feedback! I completely agree and acknowledge this limitation. The omission of the counter-narrative-only arm was driven by binding budget constraints, and I have largely revised and softened the interpretation of results throughout the paper, as will be especially apparent in the conclusion. I agree this is not a minor limitation.

The purpose of this work was to encourage other academics (ideally those with more readily available and robust research funds than myself!) to take up the questions raised here and investigate them with deserved rigor. I hope the paper is read in that spirit: not definitive or standalone evidence, but as an invitation for more empirical attention. The limitations you have identified are precisely the kinds of gaps I hope future work will fill, and I would be delighted to see others build on, challenge, or of course, even overturn these preliminary findings. I have tried to frame the contribution accordingly in the revised manuscript (including in the abstract itself), and I am grateful for feedback that has helped sharpen that framing.

R2-5: The survey design is highly transparent: participants see an advertisement from their own party attacking Trump, immediately followed by questions about perceptions of people with criminal records. The connection is obvious. This problem is particularly acute in the counter narrative first condition, where participants: (1) read about a representative complaining about the "criminal" label; (2) see the Trump advertisement using exactly that label; and (3) answer questions about perceptions of people with criminal records. The sequence essentially signals the "correct" response. The authors should have included attention checks and comprehension questions about the stimuli, as well as measures of perceived demand.

I very much understand and acknowledge the concern here, and I appreciate the critique! Attention checks were included at the beginning of the survey, though not in the middle (largely, again, as a result of budget constraints. Including an attention check in the middle or end would require siphoning the sample further rather than rejecting submissions outright or requesting returns, per Prolific’s user agreement, given the survey length.) The analysis of manipulation checks, I believe, helps (but does not completely ameliorate) these concerns. Results of these manipulation checks are described in terms of measurement in the methods section and summarized in regard to the results in the first paragraph of the “main results” section (and more conveniently, in the response to R2-12).

R2-6: The survey was fielded on July 12-13, 2025—eight months after the November 2024 election, and after Trump had already won. This completely changes the psychological context. Participants are not evaluating the advertisement as a real-time campaign message but retrospectively, with full knowledge of the election outcome. This likely introduces hindsight bias, post-election frustration or anger, and a different emotional landscape than would exist during an active campaign. The findings simply cannot be generalized to the relevant real world context of campaign messaging effects.

Agreed! This has been acknowledged in the limitations and directions for future research section. The first half of the conclusion, in fact, has been largely rewritten as a result of this (and related) comments.

R2-7: The Trump advertisement stimulus confounds visual and textual elements: participants see Trump's mugshot with the text "CONVICTED CRIMINAL" superimposed. The authors acknowledge they cannot separate these effects, but this is a substantial limitation. Mugshots themselves are stigmatizing, Trump's mugshot was widely recognized and highly iconic, and the visual element may carry independent effects. Without a condition that presents only the text or only the image, we cannot identify what drives the observed effects.

Very much understood! This was further and more prominently emphasized as a limitation (see “Additionally, as the advertisement…”), and this is precisely why the framing of the effect is the advertisement. I have thus

Attachment Submitted filename: Response to Reviewers.docx

# Peer review: Decision Letter 1

**DOI:** 10.1371/journal.pone.0355933.r003

**Authors:** Cengiz Erisen

23 Jun 2026

Dear Dr. Harney,

Thank you for submitting your manuscript to PLOS ONE. After careful consideration, we feel that it has merit but does not fully meet PLOS ONE’s publication criteria as it currently stands. Therefore, we invite you to submit a revised version of the manuscript that addresses the points raised during the review process.

Please submit your revised manuscript by Aug 07 2026 11:59PM. If you will need more time than this to complete your revisions, please reply to this message or contact the journal office at plosone@plos.org. When you're ready to submit your revision, log on to https://www.editorialmanager.com/pone/ and select the 'Submissions Needing Revision' folder to locate your manuscript file.

- A letter that responds to each point raised by the academic editor and reviewer(s). You should upload this letter as a separate file labeled 'Response to Reviewers'.
- A marked-up copy of your manuscript that highlights changes made to the original version. You should upload this as a separate file labeled 'Revised Manuscript with Track Changes'.
- An unmarked version of your revised paper without tracked changes. You should upload this as a separate file labeled 'Manuscript'.

If you would like to make changes to your financial disclosure, please include your updated statement in your cover letter. Guidelines for resubmitting your figure files are available below the reviewer comments at the end of this letter.

If applicable, we recommend that you deposit your laboratory protocols in protocols.io to enhance the reproducibility of your results. Protocols.io assigns your protocol its own identifier (DOI) so that it can be cited independently in the future. For instructions see: https://journals.plos.org/plosone/s/submission-guidelines#loc-laboratory-protocols. Additionally, PLOS ONE offers an option for publishing peer-reviewed Lab Protocol articles, which describe protocols hosted on protocols.io. Read more information on sharing protocols at https://plos.org/protocols?utm_medium=editorial-email&utm_source=authorletters&utm_campaign=protocols.

As the corresponding author, your ORCID iD is verified in the submission system and will appear in the published article. PLOS supports the use of ORCID, and we encourage all coauthors to register for an ORCID iD and use it as well. Please encourage your coauthors to verify their ORCID iD within the submission system before final acceptance, as unverified ORCID iDs will not appear in the published article. *Only*  the individual author can complete the verification step; PLOS staff *cannot*  verify ORCID iDs on behalf of authors.

We look forward to receiving your revised manuscript.

Kind regards,

Cengiz Erisen

Academic Editor

PLOS One

**Journal Requirements:**

1. If the reviewer comments include a recommendation to cite specific previously published works, please review and evaluate these publications to determine whether they are relevant and should be cited. There is no requirement to cite these works unless the editor has indicated otherwise.

2. Please review your reference list to ensure that it is complete and correct. If you have cited papers that have been retracted, please include the rationale for doing so in the manuscript text, or remove these references and replace them with relevant current references. Any changes to the reference list should be mentioned in the rebuttal letter that accompanies your revised manuscript. If you need to cite a retracted article, indicate the article’s retracted status in the References list and also include a citation and full reference for the retraction notice.

[Note: HTML markup is below. Please do not edit.]

Reviewers' comments:

Reviewer's Responses to Questions

**Comments to the Author**

Reviewer #1: All comments have been addressed

Reviewer #3: (No Response)

**********

2. Is the manuscript technically sound, and do the data support the conclusions?

Reviewer #1: Yes

Reviewer #3: Yes

**********

3. Has the statistical analysis been performed appropriately and rigorously?

Reviewer #1: Yes

Reviewer #3: Yes

**********

4. Have the authors made all data underlying the findings in their manuscript fully available?

The PLOS Data policy

Reviewer #1: Yes

Reviewer #3: Yes

**********

5. Is the manuscript presented in an intelligible fashion and written in standard English?

Reviewer #1: Yes

Reviewer #3: Yes

**********

Reviewer #1: I believe that the authors have adequately responded to my comments from the first round of reviews (though it seems like the other reviewer had more serious issues).

Reviewer #3: This is a new review of a previously revised manuscript. I read the author's responses to the original reviews and will leave it to the reviewers and editors to determine if the manuscript was appropriately revised. As the manuscript currently stands, I have two related critiques.

First, there is little discussion of the ample literature on negative campaign advertising. I realize that this manuscript is not directly interested in negative campaigning and is more interested in the effects of language (and counter-narratives). There is, however, much that can be taken from this literature, starting with work from Ansolabehere and Iyengar in the mid-1990s, on how ads like the ones emphasizing Trump's interactions with the justice system, affect people's processing of related information and their behavior. At the very least, this literature can help better answer some of the speculative questions in the discussion and concluding sections, and better inform the presentation of theory in the front end of the manuscript. I include this critique not simply to say that there are other papers that should be cited here. I include it because this experiment was conducted in the context of, and proports to help us understand, reactions to an actual negative campaign ad. The context matters. Respondents were not presented with a conversation, or a speech, or some other type of interaction; they were presented with a campaign ad used by the Democratic Party in the 2024 election. That has the potential to lead to other cognitive effects beyond the message itself that are relevant to the theoretical arguments, including what effective counter-narratives look like and how they would work in real world conditions.

Second, and perhaps more specific to an aspect of the literature, there is no attention to sophistication or related concepts. From Table 1, I assume that the survey did not include items that could be used to gauge, even rough levels of sophistication. Education is split between less than a 4-year degree and at least a 4-year degree. Education is, however, only a correlate of sophistication. I recognize that, as an experiment, the core analysis simply compares the treatment to the control (or here, the double-treatment to the single -treatment). Given the context, that's a missed opportunity, especially with outcome questions relating to political efficacy. I also recognize that, assuming these items were not included, nothing can be done empirically about this now, but it would be worth the author using the literature to speculate about what the potential effects of sophistication are here.

I also want to add that I am not an expert in modern experimental research. More specifically, I am not up on the use of platforms like Prolific. That said, the empirical analyses seem perfectly reasonable to me and the conclusions accurately reflect the results as presented.

**********

what does this mean?). If published, this will include your full peer review and any attached files.

If you choose “no”, your identity will remain anonymous but your review may still be made public.

**Do you want your identity to be public for this peer review?**  For information about this choice, including consent withdrawal, please see our Privacy Policy

Reviewer #1: **Yes:** Dan Cassino

Reviewer #3: No

**********

[NOTE: If reviewer comments were submitted as an attachment file, they will be attached to this email and accessible via the submission site. Please log into your account, locate the manuscript record, and check for the action link "View Attachments". If this link does not appear, there are no attachment files.]

To ensure your figures meet our technical requirements, please review our figure guidelines: https://journals.plos.org/plosone/s/figures

You may also use PLOS’s free figure tool, NAAS, to help you prepare publication quality figures: https://journals.plos.org/plosone/s/figures#loc-tools-for-figure-preparation.

NAAS will assess whether your figures meet our technical requirements by comparing each figure against our figure specifications.

# Author comment: Author response to Decision Letter 2

**DOI:** 10.1371/journal.pone.0355933.r004

12 Jul 2026

Dr. Erisen & Reviewers,

Thank you very much for the opportunity to provide further revisions of, “Lose the label: Messaging effects on Democrats’ perceptions of individuals with criminal records & representation.” I am grateful to you and the reviewers for the thoughtful and helpful comments which have been incorporated into these revisions.

Thank you again for your time and the opportunity to resubmit this paper again!

Reviewer #1

R1-1: I believe that the authors have adequately responded to my comments from the first round of reviews (though it seems like the other reviewer had more serious issues).

Thank you!

Reviewer #2

N/A

Reviewer #3

R3-1: This is a new review of a previously revised manuscript. I read the author's responses to the original reviews and will leave it to the reviewers and editors to determine if the manuscript was appropriately revised. As the manuscript currently stands, I have two related critiques.

First, there is little discussion of the ample literature on negative campaign advertising. I realize that this manuscript is not directly interested in negative campaigning and is more interested in the effects of language (and counter-narratives). There is, however, much that can be taken from this literature, starting with work from Ansolabehere and Iyengar in the mid-1990s, on how ads like the ones emphasizing Trump's interactions with the justice system, affect people's processing of related information and their behavior. At the very least, this literature can help better answer some of the speculative questions in the discussion and concluding sections, and better inform the presentation of theory in the front end of the manuscript. I include this critique not simply to say that there are other papers that should be cited here. I include it because this experiment was conducted in the context of, and proports to help us understand, reactions to an actual negative campaign ad. The context matters. Respondents were not presented with a conversation, or a speech, or some other type of interaction; they were presented with a campaign ad used by the Democratic Party in the 2024 election. That has the potential to lead to other cognitive effects beyond the message itself that are relevant to the theoretical arguments, including what effective counter-narratives look like and how they would work in real world conditions.

Thank you for this excellent point! The introduction and conclusion sections have now both been updated. The introduction includes a brief discussion of the negative campaign literature, while the discussion notes the importance of considering the psychological effects of negative campaign ads in the interpretation of results (and as a limitation, given the constraints on not being able to run the full factorial.) Each section’s additions have been included below:

Introduction

“The more negative angle for campaign advertisements and rhetoric is important to note: prior evidence on the effects of negative campaign advertisements suggests that such advertisements tend to be utilized less by those leading in the polls [4-5] and that Democrats tend to respond more to positive ads, compared to Republicans.[6] Additionally, meta-analytic evidence does not empirically support the use of negative campaign advertisements to aid election success.[7]”

Conclusion

This is important, as utilizing a negative campaign advertisement could moderate the effect of the counter-narrative perspective intervention, depending upon one’s personality traits, amongst other potential factors.[66]

R3-2: Second, and perhaps more specific to an aspect of the literature, there is no attention to sophistication or related concepts. From Table 1, I assume that the survey did not include items that could be used to gauge, even rough levels of sophistication. Education is split between less than a 4-year degree and at least a 4-year degree. Education is, however, only a correlate of sophistication. I recognize that, as an experiment, the core analysis simply compares the treatment to the control (or here, the double-treatment to the single -treatment). Given the context, that's a missed opportunity, especially with outcome questions relating to political efficacy. I also recognize that, assuming these items were not included, nothing can be done empirically about this now, but it would be worth the author using the literature to speculate about what the potential effects of sophistication are here.

Thank you also for making this point! Education was measured as a categorical variable with 7 categories: a) less than high school; b) high school; c) some college; d) 2-year degree; e) 4-year degree; f) master’s or higher; and g) prefer not state. Note that only one individual noted prefer not to state, and less than 10 indicated having less than a high school degree, so these values were collapsed into a single category of having a high school degree or less, which serves as the reference category. The full results table has been included Appendix B to complement the summarized Table 4 included in the main text. Most education levels were not a significant predictor of any outcome measure (using high school education or less as the reference), with exception of the advertisement support, where those with a master’s degree or higher reported significantly less support for the advertisement, on average. Table 4 has been updated with the education categories instead of the binarized version of education level.

R3-3: I also want to add that I am not an expert in modern experimental research. More specifically, I am not up on the use of platforms like Prolific. That said, the empirical analyses seem perfectly reasonable to me and the conclusions accurately reflect the results as presented.

Thank you for taking the time to review! Your effort and feedback are appreciated!

Attachment Submitted filename: Response_to_Reviewers_auresp_2.docx

# Peer review: Decision Letter 2

**DOI:** 10.1371/journal.pone.0355933.r005

**Authors:** Cengiz Erisen

28 Jul 2026

Lose the Label: Messaging Effects on Democrats’ Perceptions of Individuals with Criminal Records & Representation

PONE-D-25-63979R2

Dear Dr. Harney,

We’re pleased to inform you that your manuscript has been judged scientifically suitable for publication and will be formally accepted for publication once it meets all outstanding technical requirements.

Within one week, you’ll receive an e-mail detailing the required amendments. When these have been addressed, you’ll receive a formal acceptance letter and your manuscript will be scheduled for publication.

An invoice will be generated when your article is formally accepted. Please note, if your institution has a publishing partnership with PLOS and your article meets the relevant criteria, all or part of your publication costs will be covered. Please make sure your user information is up-to-date by logging into Editorial Manager at Editorial Manager® and clicking the ‘Update My Information' link at the top of the page. For questions related to billing, please contact billing support.

If your institution or institutions have a press office, please notify them about your upcoming paper to help maximize its impact. If they’ll be preparing press materials, please inform our press team as soon as possible -- no later than 48 hours after receiving the formal acceptance. Your manuscript will remain under strict press embargo until 2 pm Eastern Time on the date of publication. For more information, please contact onepress@plos.org.

Kind regards,

Cengiz Erisen

Academic Editor

PLOS One

Additional Editor Comments (optional):

Reviewers' comments:

Reviewer's Responses to Questions

**Comments to the Author**

Reviewer #3: All comments have been addressed

**********

2. Is the manuscript technically sound, and do the data support the conclusions?

Reviewer #3: Yes

**********

3. Has the statistical analysis been performed appropriately and rigorously?

Reviewer #3: Yes

**********

4. Have the authors made all data underlying the findings in their manuscript fully available?

The PLOS Data policy

Reviewer #3: Yes

**********

5. Is the manuscript presented in an intelligible fashion and written in standard English?

Reviewer #3: Yes

**********

Reviewer #3: (No Response)

**********

what does this mean?). If published, this will include your full peer review and any attached files.

If you choose “no”, your identity will remain anonymous but your review may still be made public.

**Do you want your identity to be public for this peer review?**  For information about this choice, including consent withdrawal, please see our Privacy Policy

Reviewer #3: No

**********

# Editor report: Acceptance letter

**DOI:** 10.1371/journal.pone.0355933.r006

**Authors:** Cengiz Erisen

PONE-D-25-63979R2

PLOS One

Dear Dr. Harney,

I'm pleased to inform you that your manuscript has been deemed suitable for publication in PLOS One. Congratulations! Your manuscript is now being handed over to our production team.

At this stage, our production department will prepare your paper for publication. This includes ensuring the following:

* All references, tables, and figures are properly cited

* All relevant supporting information is included in the manuscript submission,

* There are no issues that prevent the paper from being properly typeset

You will receive further instructions from the production team, including instructions on how to review your proof when it is ready. Please keep in mind that we are working through a large volume of accepted articles, so please give us a few days to review your paper and let you know the next and final steps.

Lastly, if your institution or institutions have a press office, please let them know about your upcoming paper now to help maximize its impact. If they'll be preparing press materials, please inform our press team within the next 48 hours. Your manuscript will remain under strict press embargo until 2 pm Eastern Time on the date of publication. For more information, please contact onepress@plos.org.

You will receive an invoice from PLOS for your publication fee after your manuscript has reached the completed accept phase. If you receive an email requesting payment before acceptance or for any other service, this may be a phishing scheme. Learn how to identify phishing emails and protect your accounts at https://explore.plos.org/phishing.

If we can help with anything else, please email us at customercare@plos.org.

Thank you for submitting your work to PLOS One and supporting open access.

Kind regards,

PLOS ONE Editorial Office Staff

on behalf of

Dr. Cengiz Erisen

Academic Editor

PLOS One
